# Detection and genomic characterisation of foot-and-mouth disease virus serotypes circulating in Cameroon using environmental sampling

**DOI:** 10.1038/s41598-024-84724-2

**Published:** 2025-01-22

**Authors:** Claire Colenutt, Andrew Shaw, Seraphine Nkie Esemu, Achah Jerome Kfusi, Bessong Willington Ojong, Emma Brown, Jemma Wadsworth, Nick J. Knowles, Donald P. King, Lucy Mande Ndip, Etienne Chevanne, Fabrizio Rosso, Keith Sumption, Simon Gubbins

**Affiliations:** 1 The Pirbright Institute, Ash Road, Pirbright, Surrey, GU24 0NF UK; 2 Laboratory for Emerging Infectious Disease, University of Buea, Buea, Cameroon; 3 European Commission for the Control of Foot-and-Mouth Disease (EuFMD), Food and Agriculture Organisation of the United Nations (FAO), Rome, Italy

**Keywords:** Virology, Viral epidemiology

## Abstract

Foot-and-mouth disease virus (FMDV) is a highly contagious, economically important disease of livestock and wildlife species. Active monitoring and understanding the epidemiology of FMDV underpin the foundations of control programmes. In many endemic areas, however, veterinary resources are limited, resulting in a requirement for simple sampling techniques to increase and supplement surveillance efforts. In this study, environmental sampling was used for the first time at livestock markets and abattoirs across Cameroon to assess the opportunities for broad scale, non-invasive disease surveillance at such sites. Environmental samples (*n* = 1994) were collected from six locations across Cameroon between May and July 2019. Concurrent with environmental sampling, a questionnaire was used to gather descriptive information on the use and practices of market and abattoir sites. Samples were screened for the presence of FMDV RNA using a pan-serotype FMDV specific real-time RT-PCR assay. Positive samples were characterised at the genomic level using next generation sequencing in combination with a novel probe-based enrichment strategy. A total of 173/1994 (8.68%) environmental samples were found to be positive for FMDV RNA. Genome length sequences were obtained from environmental samples, with phylogenetically relevant capsid sequences obtained from 14 samples, with representatives of serotypes O (*n* = 6), A (*n* = 7) and SAT 2 (*n* = 3). The questionnaire results revealed that animals in Cameroon can be transported long distances to markets and abattoirs, with varying levels of control and biosecurity practices in place. The approaches used in this study have highlighted that environmental sampling is an effective and non-invasive approach to assessing FMDV presence. Furthermore, the study has demonstrated that livestock markets, abattoirs and trucks could be targeted for the introduction of biosecurity interventions as well as providing opportunities for carrying out disease surveillance. Information resulting from such surveillance could provide valuable knowledge of circulating viruses within a region of interest, aiding strategic approaches for surveillance and control of FMDV.

**Supplementary Information:**

The online version contains supplementary material available at 10.1038/s41598-024-84724-2.

## Introduction

The control of infectious disease relies on understanding how pathogens spread within a given environment. Foot-and-mouth disease (FMD) is a highly contagious, economically important disease of cloven hooved livestock and wildlife species^1^, which has a significant impact on both food and economic security at local and international levels^2^. FMD is endemic across much of Africa, Asia and the Middle East^3^. In Cameroon, a country in West Africa which relies significantly on livestock production for both economic and food security, four antigenically distinct serotypes of foot-and-mouth disease virus (FMDV) are known to circulate: O, A, (Southern African Territories 1 (SAT 1) and Southern African Territories 2 (SAT 2)^4–7^. Within Cameroon, livestock production systems are a mix of sedentary and mobile herds^8^, with the potential for livestock to be moved over large distances and across international borders for trade^9^. Movement of animals is not formally recorded, but the cattle trade network has been described^9^ and the Adamawa Region is the major region for cattle production in Cameroon, with cattle supplied across the country from this region.

As part of the effort to control and eventually eradicate FMD^10^, the Progressive Control Pathway for Foot-and-Mouth Disease (PCP-FMD)^11^ has been developed to aid endemic countries in assessing and addressing the risks associated with FMD. The pathway provides a stepwise approach to FMD control, allowing a tailored approach based on the situation and priorities specific to a region. The initial steps of this process include investigating the circulation of FMDV and the socio-economic impact of the disease, but also identification of circulating strains and risk hot spots. Veterinary resources in countries endemic for FMD are often insufficient to manage the multiple challenges present, therefore a key challenge in addressing the risks of FMD lies in how to distribute resources appropriately. Implementing disease surveillance can be labour and resource intensive, with approaches such as serosurveillance^12^ and case finding requiring detailed knowledge of both the clinical presentation of FMD and of animal handling to collect appropriate samples. Access to appropriate laboratory facilities and the availability of suitable assays for the detection and characterisation of viruses presents additional challenges.

This study aims to establish if the use of non-invasive, environmental sampling at sites that act as hubs for livestock movement, such as livestock markets and abattoirs, could be used as a low resource demanding surveillance method for FMDV, with the end goal of providing region specific information about circulating strains of FMDV to aid disease control and decision making^13,14^. Locations across Cameroon were used to collect environmental samples as well as information about biosecurity and livestock movement practices. Combined, this can provide information both about the circulation of FMDV in a region and the risk factors that exist within the livestock market system.

Environmental sampling has been demonstrated to be an efficient method of surveillance for multiple pathogens, including SARS-CoV-2^15,16^, poliovirus^17,18^ and influenza virus^19,20^. Environmental sampling allows non-invasive sampling at herd level, avoiding the necessity of handling individual animals to collect clinical samples. Detection of FMDV RNA in both experimental and endemic settings has been demonstrated^21,22^. In this study, we aimed to expand upon the established method of environmental sampling by characterising positive environmental samples collected at livestock markets in Cameroon. We show that it is possible to identify specific strains of FMDV within environmental samples using a newly developed probe enrichment next generation sequencing (NGS) approach.

## Materials and methods

### Sampling sites

**Fig. 1:**
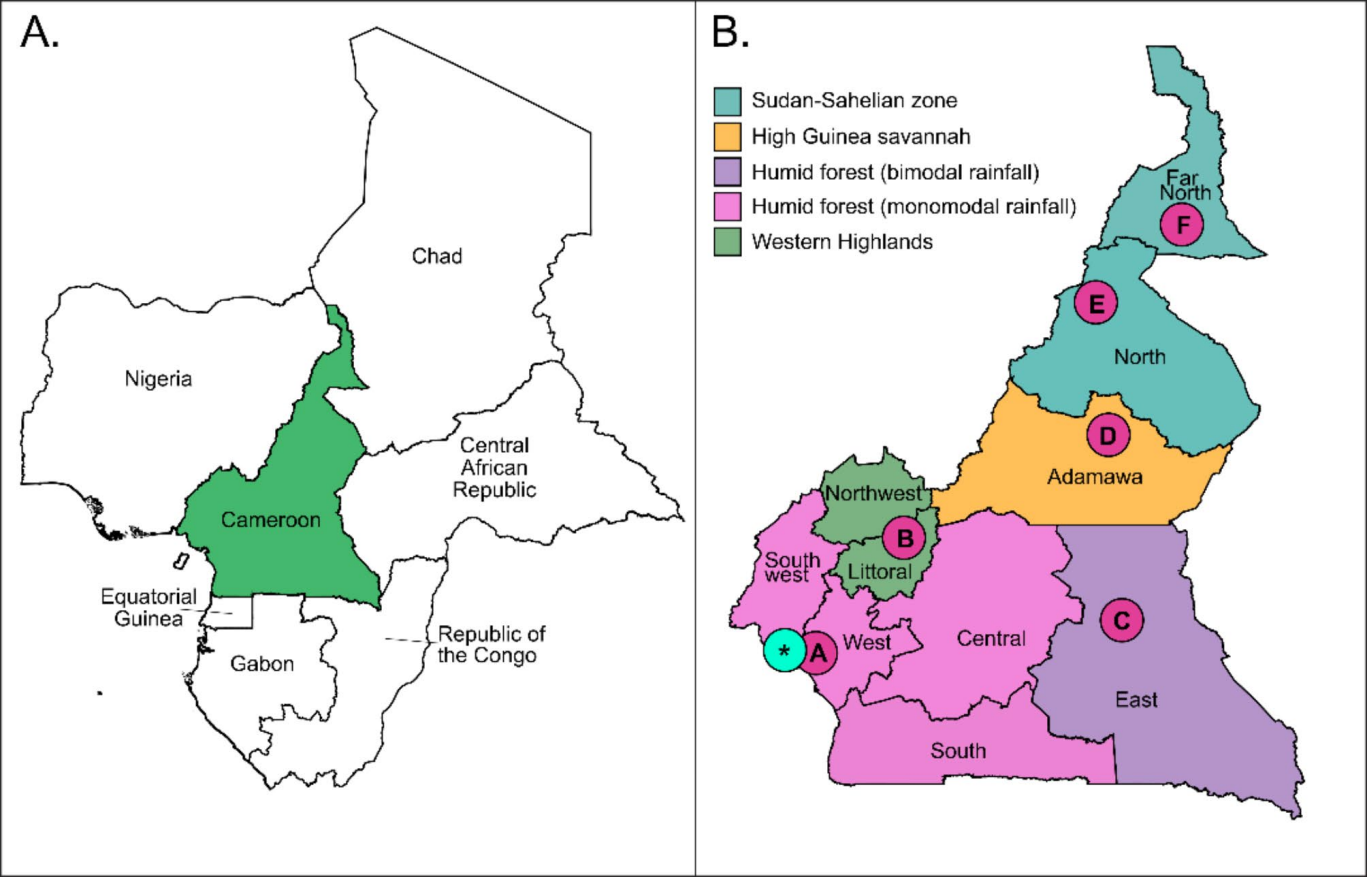
(**A**) Environmental sampling at markets and abattoirs was conducted in Cameroon, located in West Africa. (**B**) Sampling locations across Cameroon were selected to represent the different agroecological zones present in the country. A = Douala, B = Foumban, C = Bertoua, D = Ngaoundere, E = Garoua, F = Maroua. At each site, samples were collected from livestock markets and abattoirs, with the exception of Foumban (market only) and Maroua (abattoir only). All samples were transported to and stored at the Laboratory for Emerging Infectious Diseases, University of Buea (_*_) after collection.

Six locations in Cameroon (Fig. 1) were selected to represent the different agroecological zones across the country. Agroecological zones are characterised by differences in climate, vegetation and elevation and as such have corresponding differences in livestock populations^23^.

Identification of sampling sites at each location and permission to collect environmental samples from livestock markets and abattoirs was obtained with assistance from local officials. Visits to sites to collect environmental swabs were made between May and July in 2019, with multiple visits made to each site (Table 1).

**Table 1:** Environmental samples collected from each site in Cameroon.

Location	Visit date	Samples collected	Positive samples^a^	% Positive	Source of positive sample(s)^b^
**Bertoua (abattoir)**	20/06/2019	43	42	97.7	Floor
	04/07/2019	43	36	83.7	Floor
	23/07/2019	43	1	2.3	Floor
**Bertoua (market)**	6/06/2019	83	0	0	N/A
	19/06/2019	40	0	0	N/A
	04/07/2019	40	1	2.5	Fencing
	22/07/2019	40	0	0	N/A
**Douala (abattoir)**	29/05/2019	83	11	13.3	Fencing
	26/06/2019	53	17	32.1	Fencing
**Douala (market)**	12/06/2019	83	16	19.3	Truck
	26/06/2019	30	0	0	N/A
	10/07/2019	83	42	50.6	Truck
**Foumban (market)**	10/06/2019	100	2	2	Tree trunks
	24/06/2019	68	1	1.5	Water pool
	08/07/2019	83	4	4.8	Fencing, water pools
	26/07/2019	83	0	0	N/A
**Garoua (abattoir)**	11/06/2019	73	0	0	N/A
	09/07/2019	50	0	0	N/A
	23/07/2019	47	0	0	N/A
	25/07/2019	57	1	1.8	Fencing
**Garoua (market)**	11/06/2019	10	0	0	N/A
	25/06/2019	26	0	0	N/A
	09/07/2019	33	0	0	N/A
	23/07/2019	31	0	0	N/A
**Maroua (abattoir)**	12/06/2019	83	0	0	N/A
	26/06/2019	83	0	0	N/A
	10/07/2019	83	0	0	N/A
	24/07/2019	83	0	0	N/A
**Ngaoundere (abattoir)**	10/06/2019	83	0	0	N/A
	22/06/2019	23	0	0	N/A
	24/06/2019	18	0	0	N/A
	08/07/2019	33	0	0	N/A
**Ngaoundere (market)**	24/06/2019	65	0	0	N/A
	08/07/2019	50	0	0	N/A
	22/07/2019	60	0	0	N/A

a: Positive refers to a positive detection of FMDV RNA by rRT-PCR.

b: N/A = Not applicable.

The numbers of animals associated with the sites as well as general working practices were recorded using a questionnaire filled out by officials at each site. All sites were primarily used for trade and slaughter of cattle, although the market at Garoua also traded small ruminants (Table 2).

**Table 2:** Overview of information from questionnaires conducted at sampling sites. More detailed information is included in additional file 4.

Site	Samples collected	Agro-ecological zone	Frequency of use	Average number of cattle per day	Average holding time	Origin/destination of cattle	Biosecurity protocols
Bertoua abattoir	Abattoir floor	Humid forest bimodal	Daily	30	Cattle are slaughtered immediately	Adamawa, North and Far North. Transported by vehicles	Workers wear boots and coats. Facility is cleaned daily with water
Bertoua cattle market	Fencing	Humid forest bimodal	Daily	150	Up to 3 days	East, North and Far North Cameroon, Central African Republic. Transport by vehicle or by foot (local)	No biosecurity or cleaning processes reported
Douala abattoir	Abattoir floor, lairage floor and fencing	Humid forest monomodal	Daily	350	1 h	Cattle come from all over the country, transported by trucks	Workers have gloves/boots/overalls/aprons. Bimonthly cleaning and disinfection with detergent
Douala cattle market	Trucks and straw in trucks	Humid forest monomodal	Daily	1500	1–2 days	North Cameroon, Ngaoundere, Foumban, Koutaba, Adamawa. Transport over long distance is by truck	Straw from trucks delivering the cattle is burnt. No other processes reported
Foumban cattle market	Fencing, trees, water pools	Western highlands	Daily	1500	Up to 3 days	Foumban, Ngaoundere, Adamawa and North-West region. Transport is by foot (local) or by truck	No biosecurity or cleaning processes reported
Garoua abattoir	Fencing, trees	Sudano-Sahelian	Daily	50	24 h	Towns local to Garoua. Livestock transported by trekking.	Workers have coats/boots and are vaccinated against TB. Daily cleaning of facility with water.
Garoua market	Fencing, trees, feed trough	Sudano-Sahelian	Daily	100	1 day + Cattle remain until sold	Nearby towns. Transport of livestock by trekking	Market is swept weekly. Small ruminants are also traded
Maroua abattoir	Fencing, trees	Sudano-Sahelian	Daily	21	1.5 h	Localities within the Far North Region	Carcasses are burnt in trenches. Workers have boots/coats. Facility is cleaned daily with water and with bleach twice per week.
Ngaoundere abattoir	Fencing, trees, abattoir floor	High Guinea Savannah	Daily	75	2–5 h	Touborou and surrounding of Ngaoundere. Transport by trekking and trucks	Carcasses are buried, with acid added to aid decay. Workers have boots/gloves/coats/aprons. Alcohol hand disinfectant also in use. Premises are cleaned daily with water and bleach
Ngaoundere cattle market	Fencing, trees, trucks	High Guinea Savannah	Daily but main market day is Thursday	800–1000	Cattle remain at market overnight until sold	Diverse sources including Far North and localities across the Adamawa Region. Destinations include population centres in the South, West, East and Central regions of Cameroon. Transport by truck and trains	Market is swept once per week

### Sample collection

**Fig. 2:**
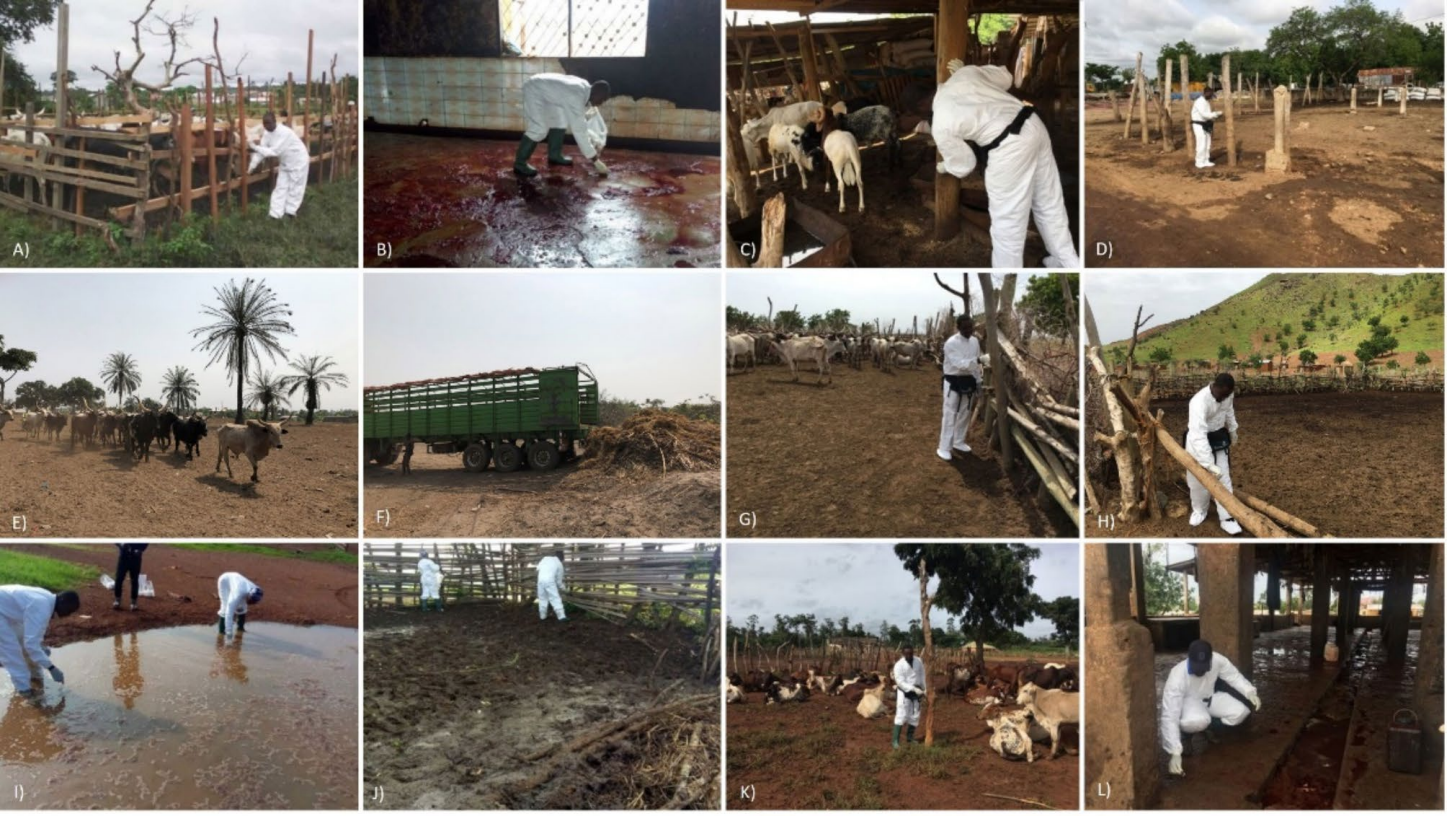
Photographs of sample collection at market and abattoir sites. (**A**) Fencing at Bertoua market. (**B**) Flooring at Bertoua abattoir. (**C**) Garoua market fencing. (**D**) Lairage fencing at Garoua abattoir. (**E**) Cattle grazing at Douala market. (**F**) Cattle transport trucks at Douala market. (**G**) Fencing at Maroua market. (**H**) Lairage fencing at Maroua abattoir. (**I**) Water pools at Foumban market. (**J**) Fencing at Foumban market. (**K**) Cattle grazing by trees at Ngaoundere market. (**L**) Sample collection from lairage floor at Ngaoundere abattoir. Informed consent obtained from all subjects and/or their legal guardian(s) for publication of identifying images in an online open-access publication.

Non-scented disposable electrostatic dust cloths (The Dustpan and Brush Store, UK), cut to an approximate size of 5 cm x 8 cm, were used to swab surfaces that were deemed likely to have had recent contact with excretions and secretions from livestock present at the facilities. Common, permanent infrastructure did not exist across all sites that would enable consistent use of surfaces for sampling between different sites. Surfaces that were selected for sampling included livestock transport trucks, trunks of trees used for shelter by cattle, drinking water pools and fencing (Fig. 2). Samples were collected at the discretion of staff from the Laboratory for Emerging Infectious Diseases, University of Buea who observed each site in use before sample collection. The same team collected samples from all sites.

After the swabbing of an area, cloths were added to 5 ml of impinger fluid (Glasgow’s minimum essential medium [Gibco, UK] with antibiotics [penicillin-streptomycin and amphotericin B {Gibco, UK}], 5% bovine serum albumin [BSA; Sigma-Aldrich, UK], and 1 M HEPES [Gibco, UK]) in a screw-top pot, and the contents were shaken to fully saturate the cloth with the medium. A disposable wooden spatula was used to remove the cloth, and at the same time, it was pressed to extract as much medium as possible. An aliquot of medium was then added directly to a guanidinium thiocyanate-based lysis buffer (catalog no. AM8500; Thermo Fisher Scientific, UK) at a sample: lysis buffer ratio of 1:2.6. Samples were then transported from sampling sites to the University of Buea for storage at + 4 °C. All samples were shipped as Category A (UN2900) infectious substances to the UK in August 2019 for processing and analysis at The Pirbright Institute. Stability of FMDV in the lysis buffer was assessed (see Additional file 1) and does not pose an issue for transport and storage of samples.

### Sample processing and analysis

Viral RNA was extracted from individual samples using the KingFisher Flex automated extraction platform (Thermo Fisher Scientific, UK) with the MagMAX viral RNA isolation kit (Thermo Fisher Scientific, UK). FMDV RNA was detected by rRT-PCR on the ABI 7500 system (Applied Biosystems, UK) using primers and probes that target the 3D polymerase region of the FMDV genome^24^.

### Genome sequencing

A probe enrichment strategy was first developed in order to achieve the necessary analytical sensitivity required to sequence FMDV RNA in environmental samples. The effectiveness of this method was assessed using a dilution series of FMDV O_1_ Manisa cell culture supernatant which was serially diluted 10-fold in negative bovine epithelium suspension. Duplicate tubes for each dilution were prepared containing 500 µl of the diluted virus. To artificially create a panel of poor-quality samples, one tube of each pair per dilution was heated to 56 °C for two hours. Total RNA was extracted from both the heated as well as paired control samples for the 10^− 2^, 10^− 4^ and 10^− 6^ dilutions using the RNeasy Mini kit (Qiagen, UK) according to the manufacturer’s instructions. First and second strand cDNA synthesis was performed as previously described^25^. Briefly, first strand synthesis was performed using the Superscript III First-Strand Synthesis System (Life Technologies) followed by treatment with RNaseH. The complementary second strand was synthesised using the NEB Second Strand Synthesis kit (New England Biolabs). Duplicate Illumina libraries were prepared using the Nextera Flex for Enrichment kit (now DNA Prep with Enrichment, Illumina) for both the control as well as heated samples. The initial, unenriched libraries were assessed with regards to mass (Qubit, Life Technologies) and size (TapeStation, Agilent). One of the library duplicates for each treatment was added to a pool and subjected to probe-based enrichment according to the manufacturer’s instructions using 10 µl of a custom panel of biotinylated oligos encompassing the complete FMDV genome (Twist Bioscience). The custom panel of 120 bp probes was designed using 622 complete genome sequences downloaded from NCBI GenBank representing all seven FMDV serotypes (Additional file 2). Probes were designed using an automatic pipeline at Twist Bioscience and were designed end-to-end along each genome. Duplicate probes were removed such that the final probe library comprised 26,275 unique probes (Additional file 2).

A final pool was made comprising each of the pre-enriched libraries as well as the pool of enriched libraries. The final pool was diluted to 4 nM and paired end reads were generated using an Illiumina MiSeq with a 2 × 150 cartridge. Following the initial experiments to evaluate the enrichment process, three sets of eight FMDV-positive environmental samples collected in Cameroon were assembled based upon both *C*_T_ value (*C*_T_ value range: 27–36) as well as epidemiological interest. The first-round libraries of each set of eight were pooled and enriched together. The three final, enriched libraries were mixed at equimolar concentration and sequenced as above.

NGS reads were assessed for quality using FASTQC, with adapters being removed using Trim Galore. The reads derived from the initial experiment using O_1_ Manisa were used for reference assembly, with the O_1_ Manisa sequence (accession number AY593823) used as a reference.

In the absence of any a priori information, the reads derived from environmental samples were initially subjected to *de novo* assembly using SPAdes^26^, with the resulting contigs identified using blastn. During the course of the analyses a contig was identified by blastn as FMDV lineage O/ME-SA/Ind-2001d. This virus lineage had not previously been reported within West Africa and, furthermore was not reported between the time of collection and time at which the samples were sequenced. We therefore reasoned that the most likely reason for the presence of Ind2001d was contamination. In order to eliminate the impact of the contamination and thus retain value from the run, reads were first mapped against the most closely related publicly available 1D (VP1 encoding) sequence using BWA-MEM v0.7.17-r1188^27^. The ‘cleaned’, unmapped reads were then used for reference assembly against west African FMDV genomes identified during the SPAdes analysis. Assemblies were manipulated using Samtools^28^, and the consensus sequence extracted using BCFtools v1.10.2^29^. Coverage depth was determined using a custom R script.

The genome-length sequences obtained using this novel method are provided in Additional file 3. However, FMDV lineages are defined based upon sequence identity within the 1D region and recombination is not observed within this section of the genome. We therefore restricted our analyses to the 1D/VP1 region of the genomes as a robust approach to identifying the serotype and lineage of viral sequences within FMDV-positive samples. VP1-coding sequences were extracted from the genome data and aligned using BioEdit v7.2.5^30^ with related and reference sequences from GenBank. Maximum Likelihood phylogenetic trees were constructed using MEGA 7^31^. The same program was used to assess the best nucleotide substitution models, which were: for type O - Tamura-Nei model Gamma distributed with Invariant sites (G + I); for type A - Tamura 3-parameter model (G); and for type SAT 2 - Hasegawa-Kishino-Yano model (G + I). VP1 sequences were submitted to GenBank and the accession numbers are shown in the trees.

### EpiCollect questionnaire

Alongside sample collection, a questionnaire was used to capture data at each sampling site in order to establish patterns of use and if any biosecurity measures were in place that would aid disease control. All information was recorded at the point of collection using EpiCollect, an open access data gathering platform (Epicollect.net).

## Results

### Detection of FMDV RNA

**Fig. 3:**
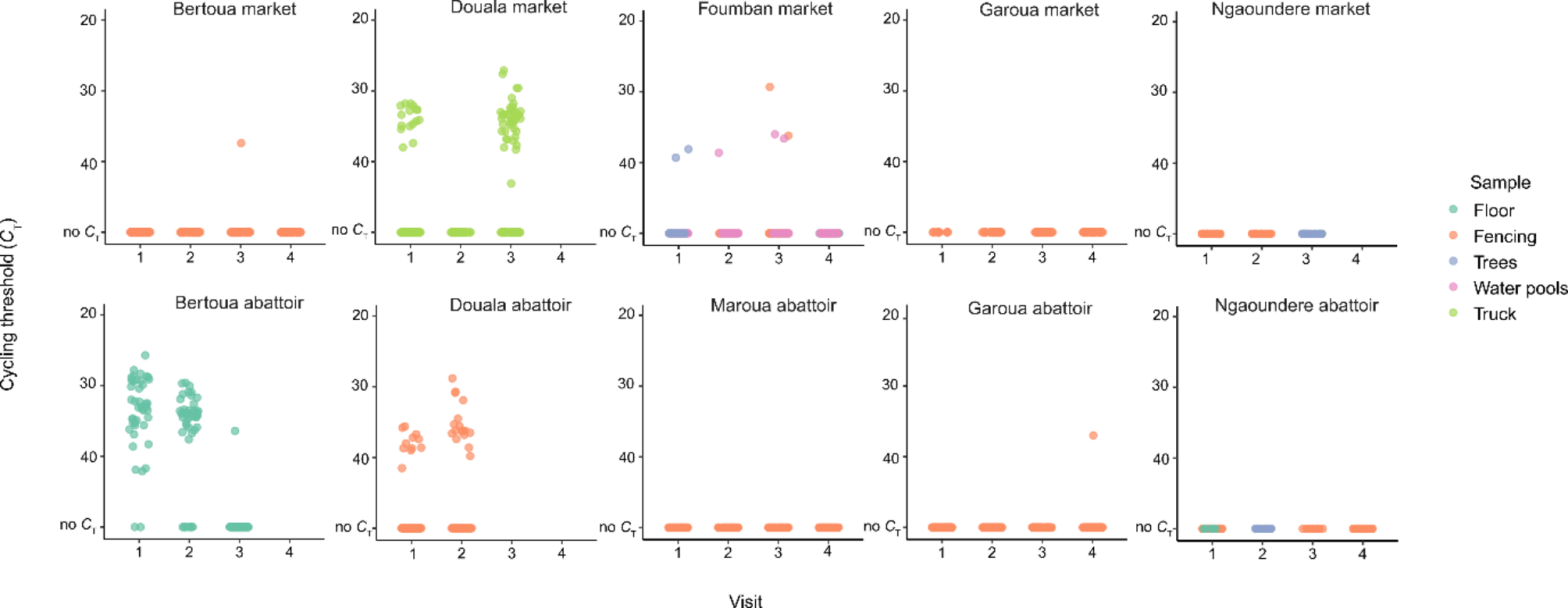
Detection of FMDV RNA in environmental samples from markets and abattoirs by rRT-PCR.

A total of 1994 samples were collected and tested for the presence of FMDV RNA. Of these, 173 samples were positive, with the majority of positive samples collected from Douala and Bertoua (Table 1; Fig. 3). Across all the sites sampled, sample types with positive FMDV RNA detection included swabs from livestock transport trucks, abattoir floors, shelter trees and water pools (Table 1). Samples containing FMDV RNA were collected at all visits made to the abattoirs at Douala and Bertoua, while at all other sites, at least one visit resulted in no detection of FMDV RNA (Fig. 3).

### Probe enrichment strategy validation

**Fig. 4:**
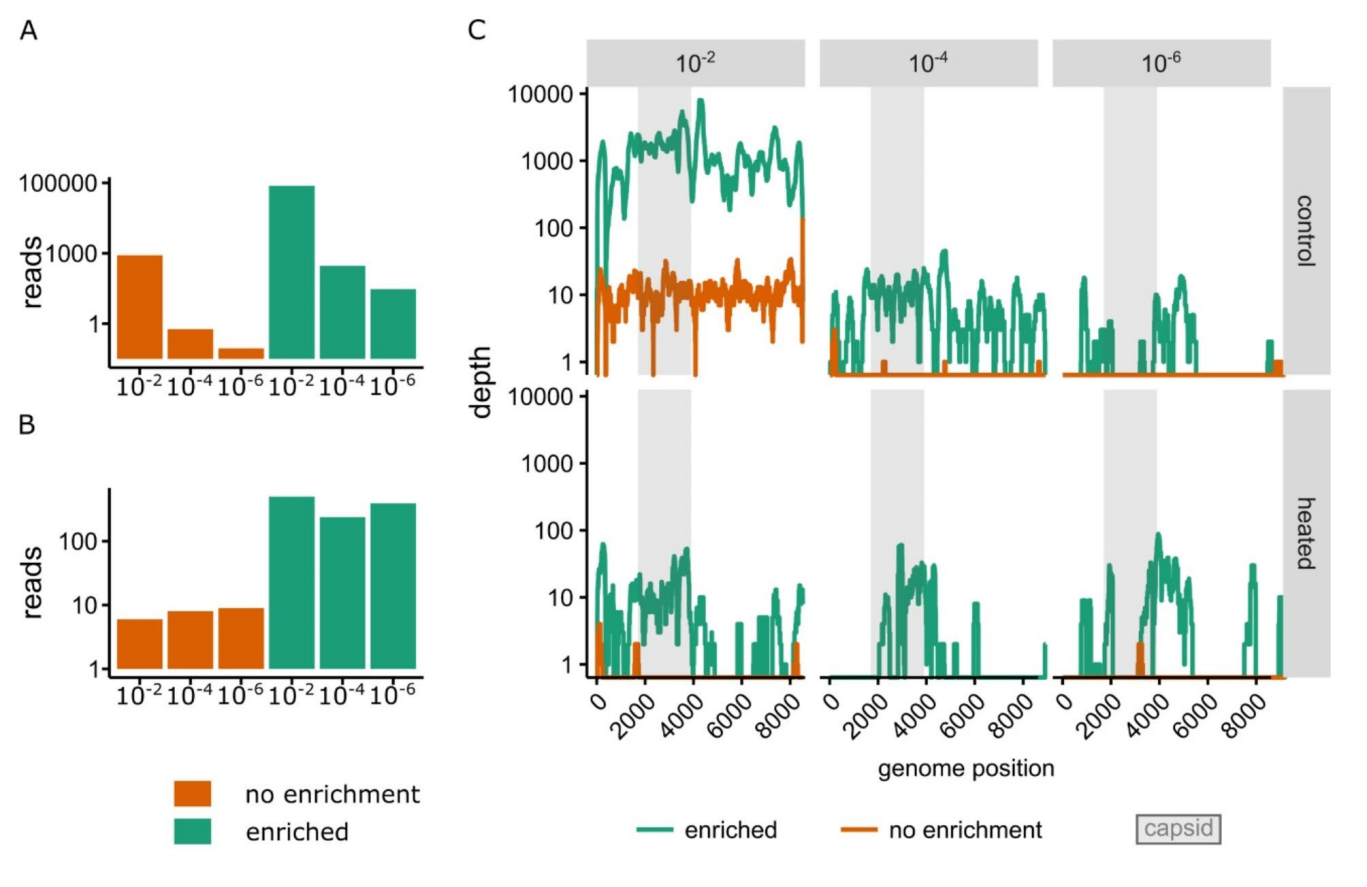
The impact of including a probe enrichment step was assessed using dilutions of O_1_ Manisa virus in epithelial suspension. In addition, one set of samples was heated to mimic poor quality samples. The inclusion of an enrichment step (green) increased the mapping rate across the genome relative ‘standard’ NGS (orange) for both control/unheated (**A**) as well as heated/poor quality (**B**) samples. Enhanced rates of mapping were observed throughout the genome (**C**), although the impact was particularly conspicuous for reads mapping to the capsid region (shaded grey).

The inclusion of a probe-based enrichment strategy was shown to increase the sensitivity of genome sequencing for FMDV (Fig. 4). In both heated (poor quality) and control samples the inclusion of an enrichment step greatly enhanced the rate of mapping. In the case of good quality (unheated) control samples, the mapping frequency across the length of the genome was increased by more than 93-fold at the 10^− 2^ dilution (Fig. 4A).

Increased rates of mapping were also observed for the 10^− 4^ and 10^− 6^ dilutions. Similarly, the inclusion of an enrichment step enhanced the mapping rates for samples which had been heated, although the rates were reduced for both enriched as well as unenriched libraries (Fig. 4B). The increase in mapping was observed throughout the genome. However, it was particularly noticeable for the capsid encoding region of P1 (Fig. 4C).

**Fig. 5:**
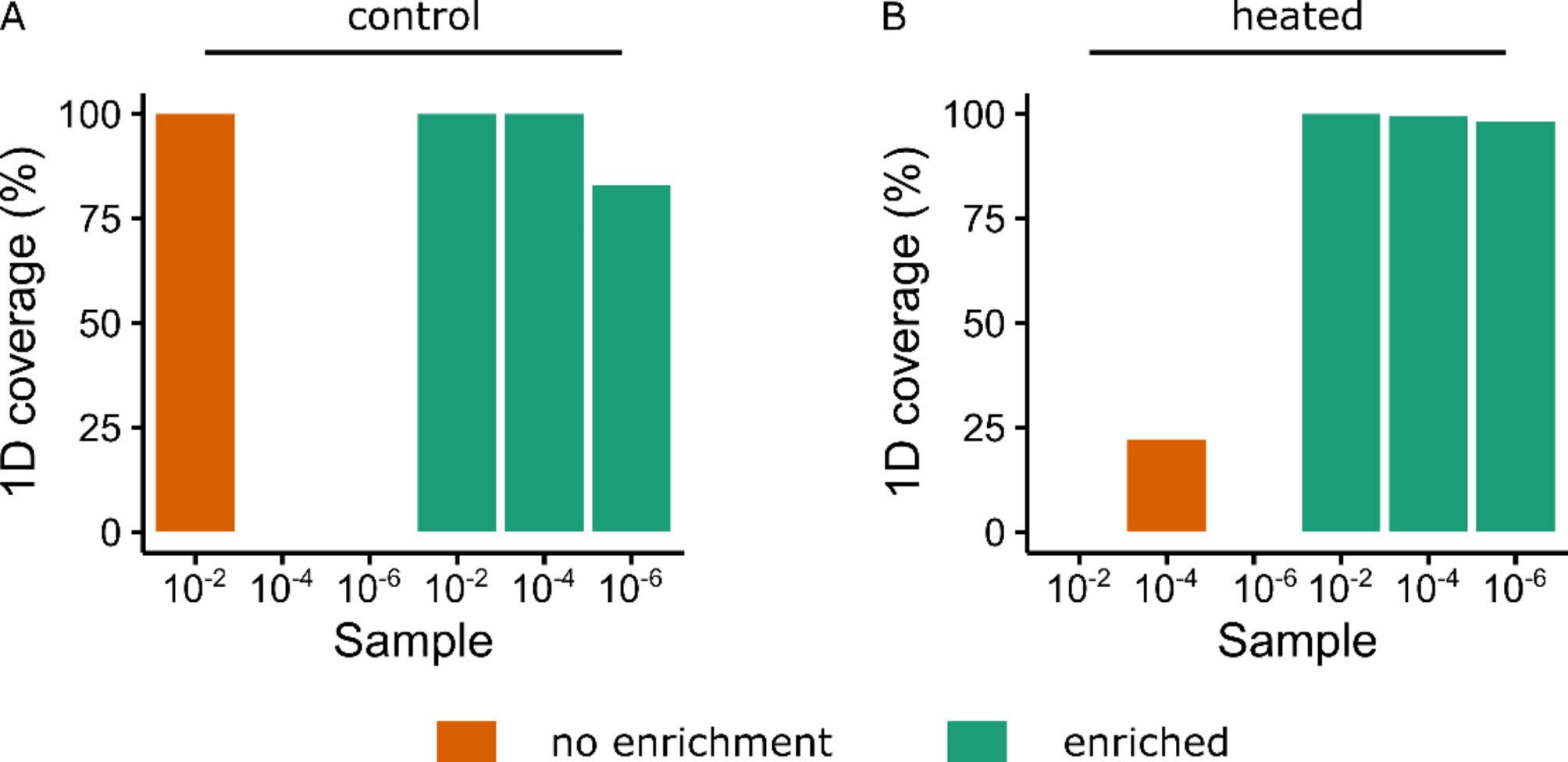
Mapping rates for the 1D region of the genome. For both control (**A**) and heated (**B**) samples, the inclusion of an enrichment step (green) allowed near 100% coverage for all of the dilutions tested. Comparatively poor rates of mapping were observed in the absence of enrichment (orange).

FMDV phylogenetic analyses are frequently based upon the 1D (VP1 encoding) region of the genome. As such, we assessed the mapping rates against 1D specifically (Fig. 5). Mapping was observed across the entire 1D region in the absence of heating at the 10^− 2^ dilution. Conspicuously, despite the absence of heating, no mapping was observed at the 10^− 4^ and 10^− 6^ dilutions for the control samples in the absence of enrichment. In contrast, the inclusion of a probe-enrichment step resulted in mapping across the majority of the 1D region for all three of the dilutions tested (Fig. 5A). The enhancement in mapping frequency was even more noticeable for poor quality samples. Samples which had been heated prior to RNA extraction showed only limited mapping against 1D in the absence of a probe enrichment step whereas the majority of 1D was covered when a probe enrichment step was incorporated (Fig. 5B).

### Sequencing environmental samples

**Fig. 6:**
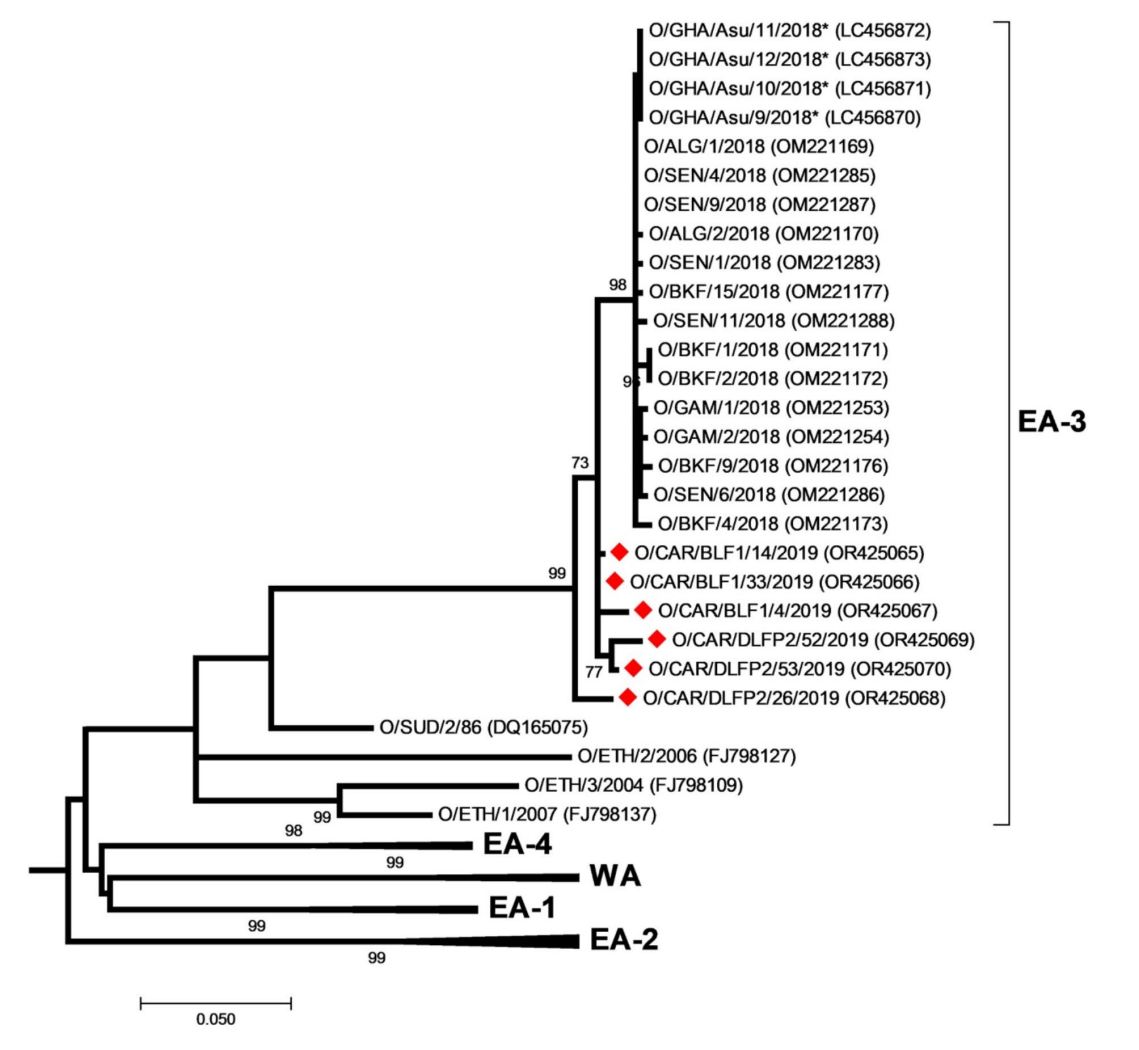
VP1 maximum likelihood phylogenetic trees for serotype O sequences generated using environmental samples. Sequences generated in this study are highlighted by red diamond symbols.

**Fig. 7:**
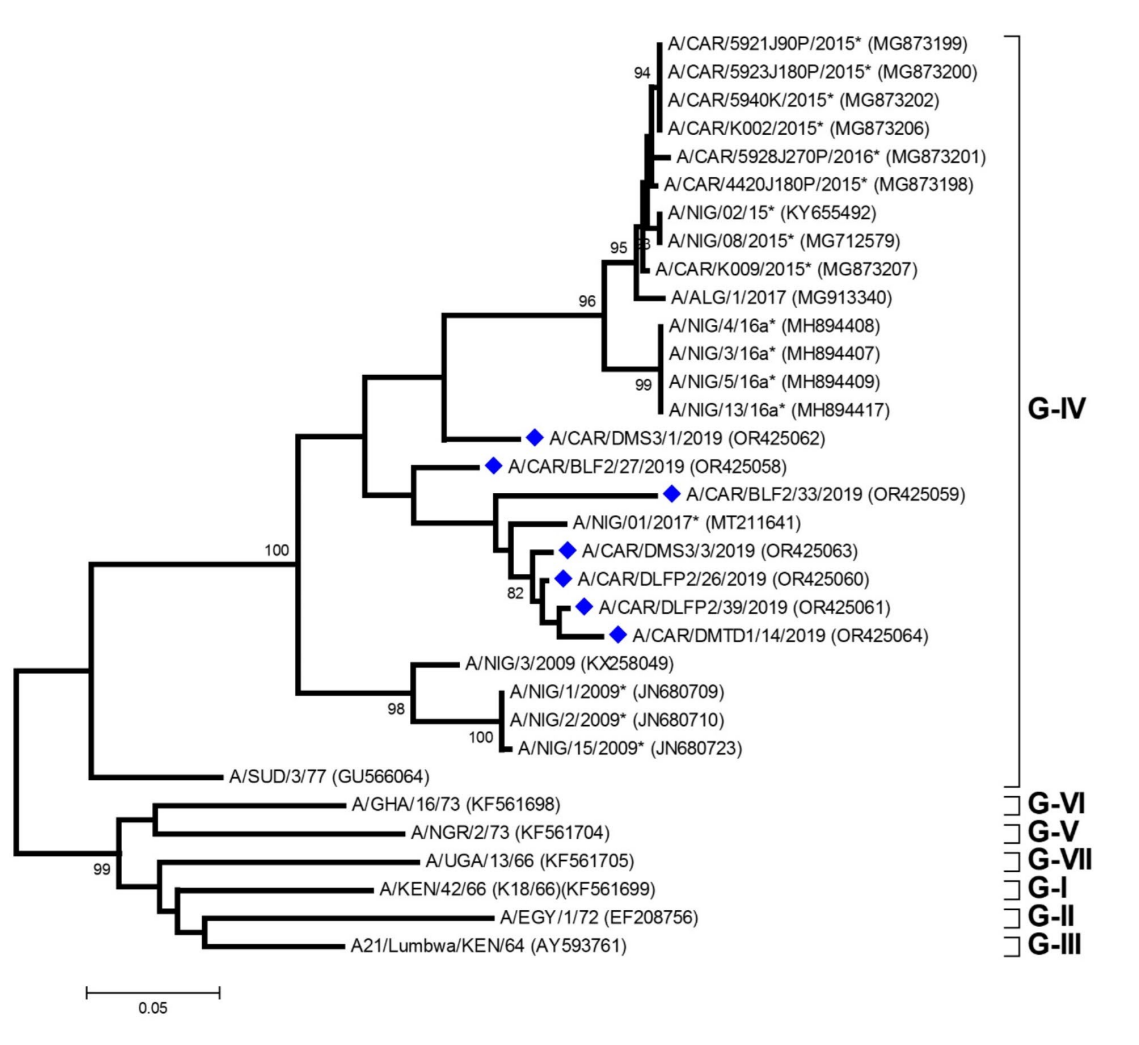
VP1 maximum likelihood phylogenetic trees for serotype A sequences generated using environmental samples. Sequences generated in this study are highlighted by blue diamond symbols.

**Fig. 8:**
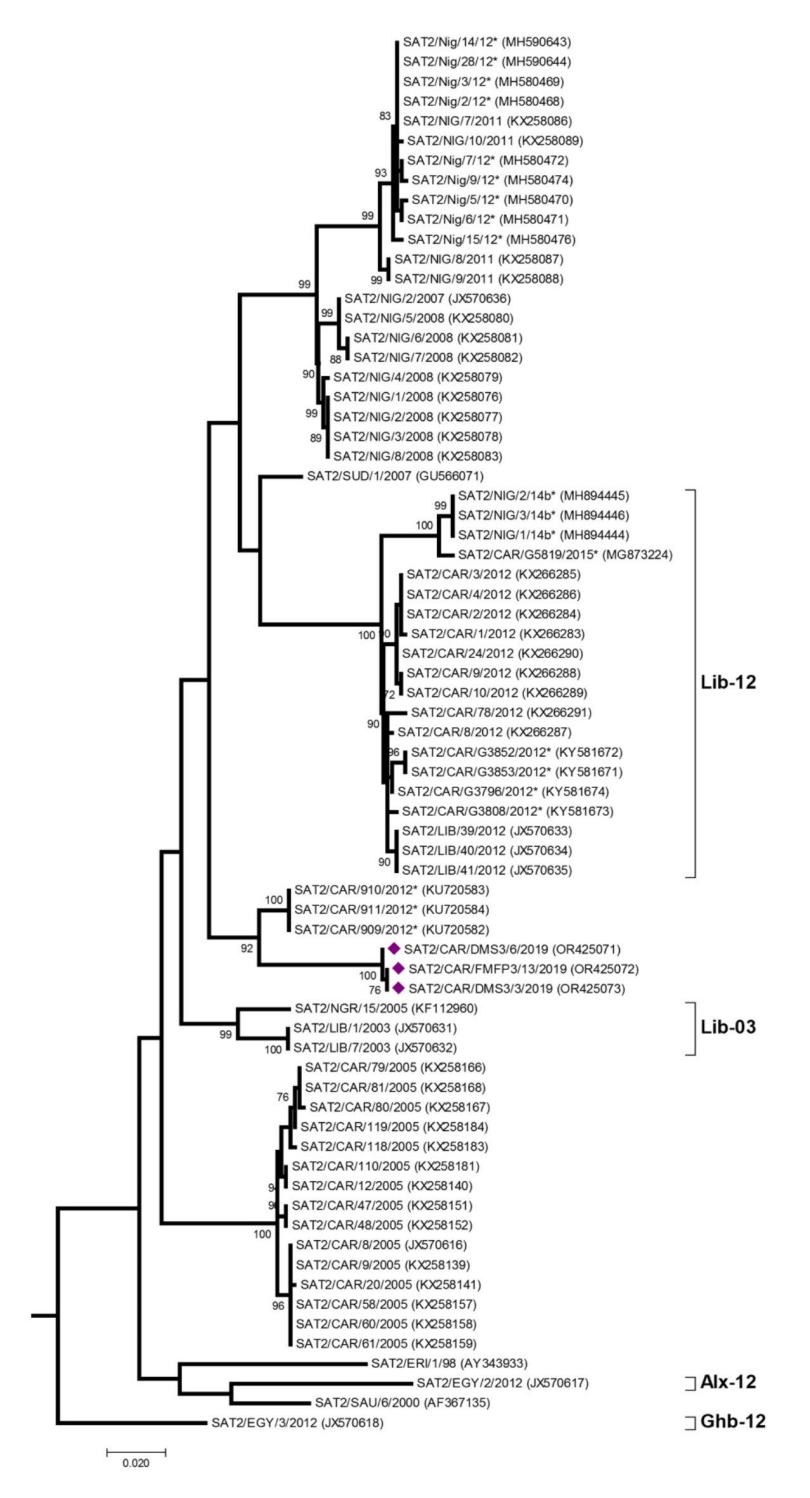
VP1 maximum likelihood phylogenetic trees for serotype SAT 2 sequences generated using environmental samples. Sequences generated in this study are highlighted by purple diamond symbols.

Genome-length sequences from environmental samples were obtained for serotypes O (Fig. 6), A (Fig. 7) and SAT 2 (Fig. 8) using probe-enriched NGS.

A total of 16 sequences were obtained from 14 samples (*C*_T_ value range: 27–35), with two samples generating sequences from different serotypes (DLFP2/26, Figures 6/7 and DMS3/3, Figures 7/8). Blastn analysis revealed these to be most closely related to viruses of West African origin, notably type O viruses from Ghana (accession number LC456870.1) and type A and SAT 2 viruses from Nigeria (accession numbers MG725872.1 and MN103523.1 respectively). The 1D region of these viruses was extracted and used to build phylogenetic trees using related isolates and lineages. The type O sequences (*n* = 6) were all representatives of the East Africa 3 (O/EA-3) topotype, with three examples each isolated in Bertoua and Douala. Seven type A virus sequences were obtained, five from Douala and two from Bertoua, all of which belonged to the genotype 4 (A/AFRICA/G-IV) topotype. Two SAT 2 sequences, both representatives of topotype VII (SAT 2/VII), were also obtained at Douala. Interestingly, a further SAT 2 sequence (also topotype VII) was obtained from samples collected at Foumban, where only 2.1% of samples were positive.

### Facility practices

Reports from sampling sites on the origin and destination of livestock demonstrate that animals can be transported over large distances and across national borders, either by trekking with livestock or transport in trucks. Distances travelled to transport livestock to market sites varied, with median distances travelled ranging from 18 km (Garoua market, quartile range 14.75-38) to 786 km (Douala market, quartile range 342–914). Distances travelled were calculated for named locations in questionnaire information by Google Maps (Table 2, Additional file 4). Biosecurity processes such as cleaning and use of disinfectant were in place for abattoirs, although disinfectant usage varied between sites. Market sites either reported no cleaning processes in place or listed occasional sweeping as a cleaning process (Table 2). Based on observations during sampling, abattoirs were more likely to have permanent infrastructure than market sites, such as buildings with solid floors (Fig. 2). The size of markets and abattoirs based on numbers of livestock varied between locations (Table 2). When considered by site type, markets showed a correlation between number of animals and positive detections of FMDV RNA (Spearman’s rho = 0.57; *P* = 0.01) but no correlation existed between numbers of livestock and positive FMDV RNA detection at abattoir sites sampled (Spearman’s rho = 0.14; *P* = 0.60).

## Discussion

This study builds on previous research, employing methods used to detect FMDV RNA in the environment on known infected farms^21^ and at a goat market in an FMDV endemic country^32^. This work demonstrates that environmental sampling provides a simple, effective and non-invasive method for the detection of FMDV RNA. In addition, methods have been developed that have enabled generation of FMDV sequence data from environmental samples.

Previously, it has been difficult to characterise FMDV in environmental samples as the content of genetic material is often of poor quality and/or low abundance, resulting in an inability to isolate and amplify live virus isolates (even following transfection), prior to sequencing. Similarly, the fragmented nature of RNA found in environmental samples abrogates the ability to amplify suitably sized amplicons by PCR for sequencing. Samples in this project were stored in lysis buffer immediately after collection, which inactivates FMDV to provide a stable storage and transport solution. This, however, prevents isolation and propagation of virus from samples, therefore requiring alternative methods to successfully carry out sequencing. In this study, the development and use of NGS probe-based enrichment methods has enabled the generation of sequence data from environmental samples, without the need to enrich the samples by virus isolation and avoiding bias from PCR amplification. This method has enabled characterisation of FMD viruses from environmental samples, increasing the value of information we can obtain from this sample type. Phylogenetic analysis of the resulting VP1 sequences demonstrated that the FMDV sequences fit with the expected serotypes and topotypes for West Africa^5^, adding to the available data on viruses circulating within the region. In line with our results, previous studies have observed that the O/EA-3, A/G-IV and SAT 2/VII lineages are the predominant FMDV lineages circulating in west Africa for serotypes O, A and SAT 2 respectively^5,7,33^. In this analysis, both serotype A and SAT 2 sequences were generated from the same sample (DMS3/3, Figs. 7 and 8), demonstrating the potential diversity of strains in livestock passing through these locations. The nature of environmental sampling means that it is impossible to determine whether the presence of two serotypes in a single sample is due to different viruses in different animals, or whether they derive from a co-infected animal^7^.

Previous studies in Cameroon have provided a picture of both the livestock trade^33^ and FMDV situation^34^, both of which are necessary to understand risk factors associated with the spread of infectious disease within livestock populations in Cameroon. Previously identified risk factors included large scale movements of animals, with cross border animal movements^35^ and mixing of livestock during transport or whilst grazing^36^. In this study, the questionnaire conducted at each of the sampling sites, and observations made during sample collections emphasised the occurrence of known risk factors for the transmission of FMDV, in particular the mixing of livestock from different locations and limited evidence of biosecurity practices at marketplaces (Table 2, Additional file 4). Marketplaces in particular lacked formal built infrastructure which would enable segregation of batches of livestock and cleaning procedures to take place. The detection of FMDV RNA in environmental samples from three of the markets in this study suggests that infected animals have passed through these locations, potentially facilitating transmission of FMDV to other animals during their time at the markets, although the time frame for presence of infectious individuals is not able to be determined from environmental sampling data. Therefore, targeting marketplaces as centres where spread of disease could be limited with biosecurity interventions is a possibility, as has been demonstrated with avian influenza^37^. Based on our results, targeted disinfection of transport vehicles may also be considered as a control measure for reducing spread of FMDV, given detections of FMDV RNA in trucks used to transport livestock to Douala market (Table 1). The sampling carried out in this study detects only FMDV RNA and makes no distinction on infectious material, but the contamination of these trucks is most likely from infectious individuals shedding material into the truck environment. Thorough cleaning of the trucks between journeys could limit exposure of susceptible livestock to contamination from previous occupants. The frequency of FMDV RNA detection correlated with the numbers of animals at market sites, with detections more likely the more animals that use the site (Table 2). At abattoirs, no correlation existed between numbers of livestock and positive detections of FMDV RNA, and this is likely due to the large amount of virus that would be shed into the environment should an infected individual be culled and butchered, limiting the importance of numbers of livestock. Use of the questionnaire to gather additional data from the sites could be expanded and improved upon in future studies. Exploring the location of these sites along value chains for livestock, detailed information of catchment areas and capturing reports of clinical FMD would inform strategic approaches for surveillance and control of FMDV.

Collection of environmental swabs varied from site to site based on availability of surfaces for sampling. For example, Douala livestock market is an open site with very little fixed infrastructure (Fig. 2), so the most appropriate surfaces for sampling were the trucks that transported cattle to the market. Positive detection of FMDV RNA in these samples implies that infectious animals were transported in the trucks, potentially indicating the movement of clinically affected livestock between locations within the country. Rather than recommending specific locations or numbers of samples for sample collection in future studies, we suggest that best practice for the collection of environmental samples is to consider interactions at each individual site, i.e., where animals (or excretions and secretions from animals) are likely to make contact with surfaces. Understanding the use and function of each location is also important in interpreting results. For example, responses to our questionnaire recorded that abattoirs in particular employed cleaning and disinfectant processes (Table 2) which would potentially reduce the chance of virus persisting in the immediate environment, depending on the effectiveness of cleaning procedures. As no sampling was carried out to determine the effectiveness of cleaning procedures in place at any of the sites, and in the context of environmental sampling, it is not possible to determine whether detection of FMDV RNA comes from recent contamination, or a build up over time. If cleaning processes were effective, it is likely that positive samples from these sites would likely be due to relatively recent contamination from infected individuals rather than a build-up of contamination over a long period of time.

Environmental sampling is a useful tool in the surveillance of infectious disease but does have associated limitations. It is important to note that environmental sampling only provides a snapshot in time of FMDV presence, with no temporal or spatial linkage of samples. For example, positive samples cannot be linked to a specific animal or outbreak location and without linked clinical data or evidence of FMDV in the region, negative environmental samples could be due to either the absence of FMDV or that FMDV was circulating, but sampling failed to detect the virus. Additionally, while the collection of samples is low cost and requires little prior knowledge of livestock diseases, the processing of samples to generate sequence data requires considerable technical expertise and expense, including specialist equipment. In this study, it was necessary for samples to be transported to the UK for analysis, rather than the analysis being carried out in Cameroon, increasing the time and expense involved in generating results. Indeed, a key objective of surveillance in an endemic setting would be to detect new ‘emerging’ strains, therefore reducing the time scales associated with processing samples is essential. Development of processing and analytical techniques, such as the use of MinION sequencing technology^38^ or other mobile detection platforms^39^ would enhance the accessibility and speed of this type of surveillance.

A specific limitation of this study is the time frame in which sampling was carried out. FMDV exhibits a seasonality in Cameroon, with more outbreaks occurring during the dry season (November to April, with some variation between agro-ecological zones), due to increased movements of livestock for grazing and access to water sources^7,40,41^. Loss of condition during this period due to lack of resources also makes livestock more susceptible to infection. All sampling for this study was carried out from May to July 2019, which falls during the wet season in Cameroon. A longer period of sampling to allow for collection of samples throughout the year could have increased the amount of data generated and explored further the patterns of FMDV in Cameroon. Inclusion of multiple sites from each agroecological zone would allow differences in climate and husbandry approaches to be assessed. Indeed, previous studies have highlighted the role of cattle breed, climate and animal movements in relation to FMDV circulation in Cameroon^7,41^. In addition, the survival and persistence of FMDV in these environments is unknown, so the time frame in which viruses may be deposited in the environment and still be detected is uncertain. General temperature profiles of the locations sampled would suggest short lived (to the scale of several days) survival/persistence in the environment^42^, although it is worth noting that other factors, such as environmental matrix, pH and relative humidity can impact viral survival. Implementation of cleaning and disinfection protocols at sites would also reduce the chances of detecting virus in samples. Further targeted surveillance studies, including the sampling of individual animals, can be undertaken guided by the results obtained using environmental sampling.

## Conclusions

To conclude, this study has demonstrated that environmental sampling at livestock markets and abattoirs provides a useful addition to surveillance tools for the detection and characterisation of FMDV. The use of environmental sampling at markets, which act as hubs for movement of livestock, provides an opportunity for carrying out surveillance work which is less resource intensive than other approaches, and has the potential to supplement current knowledge of circulating viruses within a region of interest^43^. Due to the non-clinical focus of this sampling approach, samples could be used for the detection of multiple livestock viruses, expanding the value of this sampling approach. These methods could also be expanded by assessing the feasibility of effluent sampling or sampling at communal dip tanks. Use of environmental sampling could provide a broad scale approach for surveillance of livestock disease, not only limited to FMDV but also other transboundary diseases that are the subject of control and eradication programmes^32^.

Use of environmental sampling requires no prior specialised knowledge of livestock diseases and can provide detailed additional knowledge of viruses that are circulating in a given region. The development of more sensitive sequencing protocols has increased the amount of available information from environmental samples and further developments in detection and sequencing technologies will make this type of analysis more accessible, with the potential for rapidly available results produced in local or regional laboratories.

## Electronic supplementary material

Below is the link to the electronic supplementary material.

Supplementary Material 1

Supplementary Material 2

Supplementary Material 3

Supplementary Material 4

## Data Availability

The datasets generated and analysed during the current study are available in the GenBank repository, accession numbers OR425058–OR425073.

## Abbreviations

FMD: Foot-and-mouth disease
FMDV: Foot-and-mouth disease virus
RNA: Ribonucleic acid
RT-PCR: Reverse transcription polymerase chain reaction
HEPES: 4-(2-hydroxyethyl)-1-piperazineethanesulfonic acid
