# Supplementary material for: Detection and genomic characterisation of foot-and-mouth disease virus serotypes circulating in Cameroon using environmental sampling
Source: Sci Rep. 2025 Jan 22;15:2834. doi: 10.1038/s41598-024-84724-2 (PMC11882811; doi:10.1038/s41598-024-84724-2)
Supplement: Supplementary file 1 — Supplementary Material 1 [file 41598_2024_84724_MOESM1_ESM.docx]

**Additional information 1: Lysis buffer stability**

Due to remote locations and large distances between sampling sites and the laboratory in Cameroon, temperature control during transport was not always possible. In order to ensure that samples would remain stable during transport, stability studies at a range of temperatures were performed. Two strains of FMDV (O/UKG/34/2001 and A/TAI/17/2016) were added to lysis buffer at a ratio of 1:2.6 as per use in field studies. Samples were stored at 4°C, 20°C (approximate room temperature), 30°C and 40°C for up to six months. At defined timepoints, samples were removed from experimental temperature storage and placed at -80°C until processing. Viral RNA was isolated from samples using the KingFisher Flex automated extraction platform (Thermo Fisher Scientific, UK) with the MagMAX viral RNA isolation kit (Thermo Fisher Scientific, UK). To reflect the methodology used for the detection of viral RNA in environmental samples, RNA integrity with respect to its detectability was assessed by rRT-PCR using a primer and probe set that target the 3D polymerase region of the FMDV genome (Callahan et al., 2002). FMDV RNA was stable in lysis buffer for up to three days at 30°C and two days at 40°C (Figure S1), therefore the transport and storage of samples prior to testing is not likely to have had a negative effect on the ability to detect the presence of FMDV in these samples. At room temperature (20°C) and 4°C, the stability of viral RNA is considerably longer, with detection maintained for 28 days at room temperature and up to 6 months when stored at 4°C (Figure S1).

Callahan, J. D., Brown, F., Osorio, F. A., Sur, J. H., Kramer, E., Long, G. W., Lubroth, J., Ellis, S. J., Shoulars, K. S., Gaffney, K. L., Rock, D. L., & Nelson, W. M. (2002). Use of a portable real-time reverse transcriptase-polymerase chain reaction assay for rapid detection of foot-and-mouth disease virus. *Journal of the American Veterinary Medical Association*, *220*(11), 1636–1642.


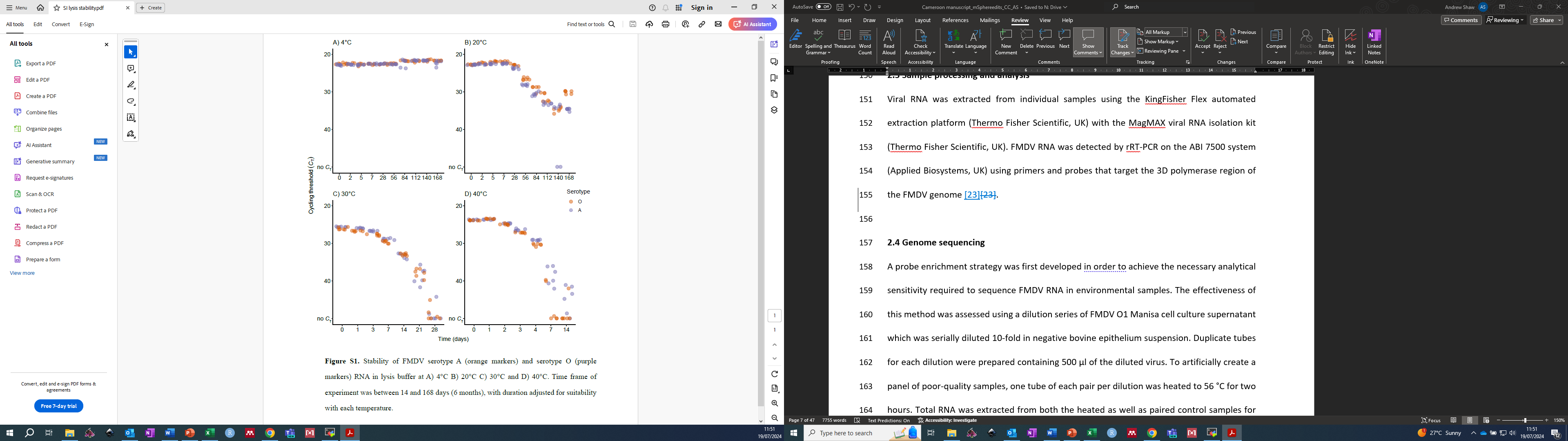


**Figure S1.** Stability of FMDV serotype A (orange markers) and serotype O (purple

markers) RNA in lysis buffer at A) 4°C B) 20°C C) 30°C and D) 40°C. Time frame of

experiment was between 14 and 168 days (6 months), with duration adjusted for suitability

with each temperature.
