# Supplementary material for: Detection and genomic characterisation of foot-and-mouth disease virus serotypes circulating in Cameroon using environmental sampling
Source: Sci Rep. 2025 Jan 22;15:2834. doi: 10.1038/s41598-024-84724-2 (PMC11882811; doi:10.1038/s41598-024-84724-2)
Supplement: Supplementary file 3 — Supplementary Material 3 [file 41598_2024_84724_MOESM3_ESM.docx]

**Additional information 3: FMDV genome sequences derived from probe-enriched data.**

The following sequences are the consensus sequences derived following reference assembly as described in the main text. These are assembled from probe enriched libraries which is not a fully validated method.

>O/CAR/BLF1/14/2019_PROBE_ENRICHMENT_CONSENSUS

TTGGTTTCTGGAGACAGGCTAAGGATGCCCTCCAGGTACCCCGAGGTAACAAGCGACACT

CGGGATCTGAGGAGGGGACTGGGACTTCTATAAAAGTGCCCGGTTTAAAAAGCTTCTATG

CCTGAATAGGTGACCGGAGGCCGGCACCTTTTCTTTTACACACAAATTACTTTATGAACA

CAACTGACTGTTTCATCGCTTTGGTACAGGCTATCAGAGAGACCAAGCTTCTGTTTAAAG

GCACTAGAAAGATGGAATTCACACTGTACAACGGTGAGAAAAAGACTTTTTACTCACGAC

CCAATCGCCACGACAACTGTTGGCTGAACACCATTTTGCAGTTGTTTAGGTATGTCGATG

AGCCATTCTTCGACTGGGTCTATAACTCGCCTGAGAATCTCACGCTTCAGGCGATTGAGC

AGCTTGAGGAGCTCACAGGTCTTAACCTGCGTGAGGGCGGACCTCCCGCCCTCGTGATTT

GGAACATCAAACACTTGTTGTACACCGGGATCGGTACTGCCTCACGACCCAGTGAGGTGT

GTATGGTCGACGGCACTGACATGTGTCTTGCTGACTTCCACGCAGGAATCTTTCTCAAAG

GCAATGAACACGCCGTGTTTGCTTGCTTGACCTCCGACGGTTGGTACGCCATCGACGACG

AGGACTTTTACCCATGGACTCCGGACCCGTCCGACGTCCTGTGTTTCGTCCCGTACGACA

TGGAACCATTCAACGGAGGCGCGGTTGTCAAAGCGACCGCGTACGTGAAGGGAGCCGGGC

AATCCAGCCCGGCTACCGGATCCCAGAACCAATCAGGCAACACGGGTAGCATAATTAACA

ACTACTACATGCAACAGTACCAGAACTCCATGGACACCCAGCTTGGGGACAACGCTATTA

GCGGAGGCTCTAATGAGGGATCCACGGACACCACCTCTACACACACAAACAACACCCAAA

ACAATGACTGGTTTTCAAAGCTGGCCAGTTCTGCTTTCAGTGGTCTTTTCGGCGCTCTTC

TCGCCGACAAGAAGACGGAGGAAACTACTCTTCTTGAAGACCGCATCCTCACCACCCGCA

ATGGGCACACAACCTCGACAACCCAGTCGAGTGTTGGAGTCACATACGGGTATGCAACAG

CTGAGGACTTTGTGAGCGGACCAAACACCTCAGGTCTCGAGACCAGAGTGGTCCAAGCAG

AACGCTTCTTCAAAACCCACCTGTTCGACTGGGTCACCAGCGACCCATTCGGACGTTGCC

ACTTGTTGGAGCTTCCAACTGACCACAAAGGTGTCTATGGCAGCCTGACCGACTCGTATG

CTTACATGCGAAACGGTTGGGATGTTGAAGTCACCGCAGTCGGGAACCAGTTCAATGGAG

GTTGCCTACTGGTGGCGATGGTGCCAGAACTCTGCTCGCTCCAGAAGAGAGAGCTGTACC

AGCTTACACTTTTCCCCCACCAGTTCATCAACCCTCGGACAAACATGACGGCACACATAA

CCGTGCCATTTGTTGGCGTCAACCGCTACGACCAGTACAAGGTGCACAAGCCCTGGACCC

TTGTGGTCATGGTCGTGGCTCCTCTGACTGTCAACAGTGAAGGTGCTCCACAAATCAAGG

TGTACGCCAACATCGCCCCCACCAACGTGCATGTCGCGGGTGAGTTCCCCTCCAAGGAGG

GGATCTTCCCAGTTGCTTGCAGTGACGGTTACGGCGGTTTGGTGACCACAGATCCAAAGA

CAGCTGATCCTGCCTACGGGAAGGTGTACAACCCTCCTCGCAACATGTTGCCAGGGCGGT

TTGTTAATCTCCTTGATGTGGCCGAGGCGTGCCCAACCTTTCTGCACTTTGAGGGCGACG

TGCCATACGTGGTCACAAAGACAGATTCAGACAGGGTGCTCACACGATTTGACCTCTCTT

TGGCAGCAAAACACATGTCAAACACCTTCTTGGCAGGTCTCGCCCAGTACTACACACAGT

ACAGCGGCACCATCAACCTGCATTTCATGTTCACAGGTCCGACTGACGCAAAAGCGCGTT

ACATGATTGCATATGCCCCTCCTGGCATGGAACCGCCTGAGACACCTGAGGCGGCTGCCC

ACTGCATTCATGCTGAATGGGACACAGGGTTGAACTCCAAATTCACATTTTCAATCCCCT

ACCTTTCAGCGGCTGATTACGCGTACACCGCGTCTGACACCGCTGAGACCACCAACGTGC

AGGGTTGGGTTTGTCTGTTCCAGATAACACACGGGAAAGCCAACGGTGACGCCTTGGTCG

TGCTGGCTAGCGCTGGCAAGGACTTCGAGCTCCGCCTACCAGTGGACGCTCGCACACAGA

CCACCTCCCCGGGTGAGTCGGCTGACCCCGTGACCGCCACCGTTGAGAACTACGGTGGTC

AAACACAAGTGCAGAGGCGCCAACACACAGATGTCGCGTTCATTCTCGACAGATTTGTGA

AAGTAACACCACAAGACCAGTTCAACGTCTTGGACCTGATGCAGGCCCCTGCACACACGC

TGGTTGGGGCGCTCCTTCGCACTGCTACTTACTACTTCGCAGATTTAGAAGTGGCAGTGA

AGCACGAGGGGAACCTCACGTGGGTCCCCAACGGGGCGCCAGAGACAGCTCTGGACAACA

CCACCAACCCAACAGCATACCACAAGGCACCACTCACCCGGCTCGCCCTGCCCTACACAG

CACCACACCGCGTGCTTGCAACCGTTTACAACGGATCTTGCAAATACGGTGGGGCCCCGG

TGACCAACGTGAGAGGTGACCTTCAGGTGTTGGCACAGAAGGCAGCTCGGAATTTGCCTA

CTTCTTTCAACTACGGTGCCATCAAAGCCACCCGGGTGACTGAACTGCTTTACCGCATGA

AGAGGGCCGAGACATACTGCCCCCGGCCCCTGCTGGCAATTCACCCGACAGAGGCCAGAC

ACAAACAGAAGATAGTGGCACCTGTGAAACAACTTCTAAACTTTGACCTACTTAAGTTGG

CAGGGGACGTCGAGTCCAACCCTGGACCCTTCTTCTTCTCCGACGTTAGGTCGAACTTCA

CCAAGCTGGTGGAGACCATTGGCAAAATGCAGGACGACATGGCAACAAAACACGGACCCG

ACTTTAACAGGTTGGTGTCCGCATTTGAGGAACTAGCCACTGGAGTGAAAGCCATCAGGA

CTGGCCTCGACGAGGCCAAACCGTGGTACAAGCTTGTCAAACTCCTGAGCCGCCTGTCGT

GCATGGCCGTTGCAGCAGCACGGTCAAAGGACCCTGTCCTTGTGGCCATCATGCTGGCTG

ACACCGGCCTTGAGATCCTGGACAGCACCTTTGTTGTGAAAAAGATCTCCGACTCGCTCT

CCGGTCTCTTCCACGTGCCGGCCCCCGTCTTCAGTTTCGGAGCCCCGATTCTGCTGGCCG

GACTGGTCAAAGTTGCCTCAAGTTTCTTTCGGTCCACACCTGAGGACCTTGAGAGAGCGG

AGAAGCAGCTCAAAGCACGTGACATCAACGACATATTCGCCATTCTCAAGAACGGCGAGT

GGCTGGTCAAACTGATCCTCGCTATCCGCGACTGGGTAAAAGCGTGGATTGCCTCAGAAG

AGAAGTTCGTTGCCGTGACGGACTTGGTGCCTGACATCCTTGAAAAGCAGCGGGACCTCA

ACGACCCTAGCAAATACAAGGAGGCCAAGGAGTGGCTCGACAACGCGCGTCAGACGTGCT

TGAAGAGCGGCAACATTCATATTGCCAGCCTGTGCAAGGTCACAGCCCCAGCACCCAGCA

GGTCGAGACCAGAACCCGTGGTTGTTTGCCTCCGCGGCAAATCAGGCCAGGGGAAAAGTT

TCCTTGCGAACGTGCTCGCACAGGCAATCTCCACACATTTTACTGGCAGAACAGACTCTG

TCTGGTACTGCCCGCCTGACCCTGACCACTTCGACGGCTACAACCAACAGACCGTTGTTG

TGATGGATGATTTGGGCCAGAACCCCGACGGCAAAGACTTCAAGTACTTTGCCCAGATGG

TTTCAACTACGGGGTTCATCCCGCCCATGGCCTCACTAGAAGACAAGGGCAAGCCTTTCA

ACAGCAAGGTCATCATCGCTACCACCAATCTGTACTCAGGGTTTACCCCGAGGACCATGG

TGTGCCCTGATGCGCTGAACCGAAGGTTTCACTTTGACATTGACGTGAGTGCCAAGGACG

GGTACAAAATTAACAACAAATTGGACATAATCAAAGCTCTTGAAGACACACACACCAACC

CAGTGGCAATGTTCCAGTACGATTGTGCCCTTCTCAACGGCATGGCCGTTGAAATGAAGA

GAATGCAACAAGATCTTTTCAATCCCCAACAGCCCCTCCAGAGCGTTTACCAACTCGTTC

AGGAGGTGATTGATCGGGTCGAGCACCACGAGAAAGTGGCAAGTCACCCGATTTTCAAGC

AGATCTCAATTCCTTCCCAAAAGTCCGTGCTCTACTTTCTCATCGAGAAGGGCCAGCACG

AAGCTGCAATTGAGTTCTTCGAAGGGATGGTCAGTGACCCCATCAAGGAGGAACTGCGCC

CCCTTCTTCAACACACCTCATTCGTGAAACGCGCTTTCAAGCGGCTGAAGGAAAACTTTG

AGATTGTTGCCCTGGTTTTGGCCCTTCTGGCCAACATCATCATCATGATCCGCGAGACCC

ACAAGAGACAGGAAATGGTGGACGCCGCCGTGAACGACTACATCGAACGCGCCGGTATCA

CCACCGATGACCAGACTCTTGATGAAGCGGAAAAGAACCCCCTGGAGACAACTGGTGCCA

GCACCGTTGGTTTCAGGGAGAGAACTCCCCCGCAGAAGGCAGGCGATGACGTGAACTCCG

AGCCTGTCAACTCCACGGGGAAAGAACCACAAGCTGAAGGACCCTACGCCGGACCGATGG

AGCGCCAGAAACCTCTGAAAGTGAAAACCAAATTACCACTGCAGGAGGGGCCCTACGCCG

GACCGATGGAGAGACAACAACCACTGAAGGTGAAAGTGAAGCCCCCCGTCGTGAAAGAAG

GACCTTACGAGGGGCCAGTGAAGAAACCTGTCGCTTTGAAAGTGAAAGCCAAGAACATGA

TTATCACGGAGAGTGGTGCACCGCCCACCGACTTGCAAAAGATGGTGATGGCCAACACCA

AGCCGGTCGAGCTCATACTCGACGGGAAGACAGTGGCAATCTGCTGTGCTACTGGAGTGT

TTGGGACTGCCTATCTCGTGCCTCGTCATCTTTTCGCTGAGAAGTATGACAAGATCATGA

TTGACGGCAGAGCCCTGACAGACCGTGATTTTAGAGTGTTTGAATTTGAGATTAAAGTAA

AAGGACAGGACATGCTCTCAGACGCCGCACTCATGGTGCTGCACCGTGGGAACCGCGTGA

GAGACATCACGAAACACTTTCGTGATCAAGCAAGAATGAGGAAAGGAACCCCCGTCGTTG

GTGTAATCAACAACGCCGACGTCGGGAGACTCATCTTCTCTGGAGAGGCACTCACCTACA

AAGACATTGTGGTGTGTATGGACGGTGACACCATGCCAGGCCTCTTTGCCTACAGAGCCG

CCACCAAGGCTGGCTACTGTGGAGGAGCCGTTCTCGCGAAGGACGGAGCCGAGACGTTCA

TCGTCGGCACTCACTCCGCAGGAGGGAATGGAGTTGGTTACTGCTCATGTGTTTCCAGGT

CCATGCTCCAGCAAATGAAGGCACACGTCGACCCTGAGCCACACCATGAAGGGTTGATCG

TGGACACCAGAGATGTGGAAGAGCGCGTGCATGTCATGCGCAAAACCAAGTTGGCACCCA

CCGTCGCGCACGGTGTGTTCAACCCCGACTTCGGGCCAGCTGCACTGTCTAACAACGACA

AGCGCCTGAGTGAGGGGGTTGTCCTCGACGACGTCATCTTCTCCAAACACAAGGGAGACA

CGAAGATGTCAGAGGCAGACAAGAAGCTGTTCAGACTTTGTGCCGCTGACTACGCGTCGC

ACCTGCACTCAGTTCTTGGTACAGCAAACGCCCCATTGAGCATCTACGAGGCCATCAAGG

GCATCGACGGACTCGATGCCATGGAGCCCGACACCGCTCCGGGTTTGCCCTGGGCCACCC

AGGGGAAACGCCGTGGAGCCCTCATTGATTTCGAGAACGGCACCGTCGGACCCGAAGTTG

AAGAGGCACTGAAGCTCATGGAGAAAAGAGAGTACAAGTTCACATGTCAGACTTTCCTGA

AGGACGAGATTCGTCCGTTGGAAAAGGTTCGTGCCGGCAAGACTCGCATTGTCGACGTCC

TGCCCGTTGAGCATATTCTTTACACCAGGATGATGATTGGCAAGTTTTGTGCACAAATGC

ACTCCAACAACGGACCGCTCATTGGCTCTGCGGTCGGTTGCAATCCAGATGTTGATTGGC

AGAGATTTGGAACCCACTTTGCCCAGTATAGGAACGTGTGGGACGTGGATTATTCGGCCT

TCGATGCTAACCACTGTAGTGACGCCATGAACATCATGTTCGAAGAGGTCTTCCGTCCAG

AATTCGGTTTCCACCCCAACGCAGAGTGGATTCTGAAGACTCTGGTCAACACGGAGCACG

CCTATGAGAACAAACGCACTACAGTGGAAGGTGGTATGCCCTCAGGCTGCTCCGCCACCA

GCATCATCAACACAATCCTCAACAACATTTACGTGCTCTACGCCCTGCGTAGACACTATG

AGGGAGTCGAGCTGTCTACCTACTCGATGATTTCCTACGGGGATGACATCGTGGTGGCAA

GTGATTTTGACCTGGATTTTGAGGCCCTCAAGCCTCACTTCAAGTCCGTTGGCCAAACCA

TCACTCCAGCCGACAAAAGTGACAAAGGTTTTGTTCTTGGTCAGTCCATTACTGATGTTA

CTTTCCTCAAGAGACACTTCCACATGGATTATGGAACTGGGTTTTACAAACCTGTGATGG

CTTCGAAGACCCTCGAGGCTATCCTCTCCTTTGCACGCCGTGGGACCATACAGGAGAAGT

TGATCTCCGTGGCAGGACTCGCCGTCCACTCTGGGCCTAGCGAGTACCAGAGACTCTTTG

AGCCTTTCACTGGGCTCTTTGAGATCCCTAGCTACAGATCACTTTACCTGCGTTGGGTGA

ACGCCGTGTGCGGTGACGCATAATCCCCTAGCTGACAATTGGCAAAAGTTGCTAGGGCGA

GTGTTGCCGTAGGAGTGAAAAGCCCGAAAGGGCTTTTTCCCGCTTCCTAAATTACAAAAA

AAAAA

>A/CAR/DMS3/3/2019_PROBE_ENRICHMENT_CONSENSUS

CTTCCCAATTTTAAAAAAAAATTGGGAAGCGGGTAAGGCTTTTCAGCCTTTCACTCCTAC

GGCGACACTCGCCTCAACAGCCTTGCCAATTGAAGTTGAGGGATTATGCGTCGCCGCACA

CGGCGTTCACCCAACGCAGGTAAAGTGATCTGTAGCTGGGGATCTCGAAAAGCCCGGTAA

AAGGCTCAAAGAGTCTCCGGTACTCGTCAGGTCCTGAGTGGACGGCGAGTCCTGCCACGG

AGATCAACTTCTCCTGTATGGTCCCACGGCGTGCAAAGGAGAGGATAGCTTCAAGGGTCT

TTGAAGCCATCACAGGTTTGTAAAACCCAGTTCCGTAATCCATGTGGAAGTGCCTCTTGA

GGAAAGTGACGTCAGTGATGGACTGACCAAGAACAAAACCTTTGTCACTCTTGTCGGCTG

GAGTGATAGTTTGACCAACGGACTTGAAGTGAGGCTTGAGAGCTTCAAAGTCCAAATCAA

AATCACTTGCCACCACGATGTCATCCCCGTAGGAAATCATCGAGTAGGTAGACAGCTCGA

CTCCCTCATAGTGTCTACGCAGGGCGTAGAGCACGTAAATGTTGTTGAGAATTGTGTTGA

TGATGCTGGTAGCGGAACAGCCCGAGGGCATACCGCCTTCCACAGTGATGCGCTTGTTCT

CGTAGGCGTGCTCCGTGTTGACCAGAGTTTTCAGGATCCACTCTGCGTTGGGGTGGAAGC

CGAATTCCGGACGGAACACCTCCTCAAACATGATGTTCATAGCGTCACTGCAGTGGTTGG

CATCAAAGGCCGAATAGTCCACATCCCACACGTTTCTGTACTGGGCAAAATGGGTTCCAA

ATCTCTGCCAATCAACATCTGGGTTGCAACCGACCGCCGAGCCAATTAGCGGTCCGTTGT

TGGAGTGCATCTGTGCACAAAATTTGCCAATCATCATTCTGGTGTAAAGAATATGTTCAA

CAGGCAGGACGTCAACGATGCGAGTCTTGCCGGCACGAACTTTTCCCATCGGGCGTATTT

CGTCCTTCAGGAATGTTTGACATGTGAACTTGTACTCCCTTTTCTCCATGAGCTTCAGTG

CCTCTTCAATCTCGGGCCCGACAGTGCCGTTCTCGAAATCAATAAGGGCTCCACGGCGCT

TTCCCTGGGTGGCCCAGGGCAAACCTGGAGCGGTGTCAGGCTCCATAGCGTCGAGTCCGT

CGACGCCCTTGATAGCCTCGTAGATGCTCAATGGGGCATTCGCTGTACCAAGAACTGAGT

GCAGGTGCGACGCGTAGTCGGCGGCACAGAGCCTGAACAGCTTCTTATCCTCTTCCGACA

TCTTCGTGTCTCCCTTGTGTTTGGAGAAGATAACATCGTCGAGGACAACTCCATCATTCA

GGCGTTTGTCGTTGTTGGACAACGCGGCCGGCCCAAAGTCAGGGTTGAACACACCGTGCG

CGACGGTGGGTGCAAGCTTAGTTTTGCGCATGACGTGCACGCGCTCTTCCACATCTCTGG

TATCCACAATCAACCCCTCGTGGTGTGGTTCAGGGTCAACGTGTGCCTTCATTTGTAAGA

GCATGGACTTGGAGACGCACGAGCAGTAACCAACTCCATTCCCTCCCGCGGAGTGAGTGC

CGACGATGAAAGTCTCGGCTCCGTCTTTTGCAAGAACGGCTCCCCCGCAGTAGCCAGCCT

TGGTGGCGGCTCTGTAGGCAAAAAGGCCTGGCATGGTGTCACCATCCATACACACTACAA

TGTCTTTGTAGGTGAGCGCCTCTCCAGAGAAAATGAGTCTCCCAACGTCAGCATTGTTGA

CTACGCCAACGACGGGGGTTCCCTTTCTCATCTTTGCTTGATCACGAAAGTGTTTCGTGA

TGTCTCTCACTCGGTTCCCACGGTGTAACACCATGAGAGCGGCATCTGAGATCATGTCCT

GTCCTTTTACTCTAATCTCAAACTCAAACACTCTAAAATCACGGTCTGTCATGGCTCTGC

CGTCAAGCATAATCTTGTCATACTTCTCAGCGAAAAGATGGCGAGGCACAAGATAGGCAG

TGCCGAACACTCCAGTGGCACAGCAGATTGCCACTGTCTTCCCGTCGAGAATGAGCTCGA

CCGGCTTGGTGTTGGCCATCACCATCTTTTGCAAGTCGGTGGGTGGCGCTCCACTTTCCG

TGATAATCACGTTCTTGGCTTTCACTTTCAAAGCGACAGGTTTCTTCATTGGCCCCTCGT

AAGGTCCTTCCTTCACGACGGGGTTCTTCACTTTAACCTTCAGTGGTTGTTGTCTCTCCA

TCGGACCGGCGTAAGGTCCCTCCGGTCGTGGCAGCTTGGCTTTCACTTTCAGGGGTTTCT

GACGCTCCATCGGCCCAACGTAGGGTCCTTCAGCTTGTGGTTTCTCCTCCAAGGGTTTGG

CAGGCTCGGTGTTCCCGTCATCGTCTGCTTTCTGCCCGGGGAGGACTCTCTCCCTAAACC

CAACGGTACTGGAACCGGTTGTTTCTAGGGGGTTCTTCTCTGCTTCATCCAGAGTCTTAT

CATCGGTGGTAATGCCGGCGCGTTCGATGTAGTCGTTCACAGCGGCGTCCACCATTTCCT

GTCTCTTGTGGGTCTCGCGGACCGTGATGATGATGTTGGCCAGAAGAGCCAGCACCAGGG

CAACAATTTCAAAATTTTCCTTCAGGCGCTTAAAAGCGCGTTTCACGAATGAGGTGTGTT

GGAGGAGAGGGCGCAGTTCCTCCTTCAGGGAGTCACTGACCATTCCTTCAAAGAATTCAA

TTGCAGCTTCGTGCTGTCCCTTCTCAATGAGAAAGTAAAGCACGGACTTTTGGGAAGGAA

TTGAGATTTGCTTGAAAATGGGGTGGCTCGCCACTTTCTCGTGGTGTTCGACCCGCTCAA

TCACCTCCTGAACAAGCTGGTAAACGTTCTGGAGGGGCTGTTGAGGATTGAAAACATCTT

GTTGCAGTCTCTTCATTTCAACGGCCATGCCGTTGAGAAGGGCACAATCGTACTGAAACA

TTGCCACTGGGTTGGTGTGCGTGTCTTCAAGAGCTTTGATTATATCCAATCTGTTATTTA

TTTTGTACCCATCCTTGGCACTCACGTCGATGTCAAAGTGGAACCTTCGGTTCAGTGCGT

CGGGGCACACCATAGTCCTCGGGGTAAAACCCGAGTACAAGTTGGTGGTGGCGATGATGA

CCTTACTGTTGAAAGGTTTGCCCTTGTCTTCTAGTGATGCCATGGGCGGGATGAATCCCG

TGGTTGAGACCATCTGGGCAAAGTACTTGAAGTCCTTGCCGTCGGGGTTCTGGCCCAAAT

CATCCATTACAACAACGGTCTGCTGGTTGTAACCGTCAAAGTGGTCAGGGTCAGGCGGGC

AGTACCAAACTGAGTCCGTTCTGCCGGTGAAGTGTGTGGAGATTGCTTGTGCAAGCACGT

TCGCAAGGAAACTCTTCCCTTGGCCAGATCTGCCACGGAGACAGACGACCACGGGTTCCG

GTCTCGACCTGCTGGGTGCTGGGGCAGTCACTTTGCACAGGTTGGCGATGTGAGTGTTCC

CGTTCTTCAGACACGCTTGGCGCGCGTTGTCGAGCCACTCCTTGGCTTCCTTGTACTTGC

TAGGGTCGTTTAAGTCCCGTTGCTTTTCAAGGATGCCAGGCACCAGGTCCGTCATGGTGA

CGAACTTTTCTTCTGAGGCGATCCATGCCTTTATCCAGTCGCGGATGGCAAGGATCAGTT

TGACCAGCCACTCGCCATTCTTGAGAATGGCGAATATGTCGTTGATGTCACGTGCTTTGA

GCTGTTTTTCTGCTCTCTCAAGATCCTCGGGCGTGGACCGGAAGAAACTTGAGGCGACCT

TGACCAACCCGGCTAACAGAACCGGAGCCCCGAAACTGAAAACGGGGGCCGGCACGTGAA

AGAGACTGGAGAGCGAGTCGGAGATTTTCTTCACGACGAAAGTGCTGTCCAGAATCTCAA

GGCCGGTGTCAGCTAGCATGATGGCCACAAGGACTGGGTCCTTTGACCGTGCTGCTACAG

CGGCCATGCACGACAGGCGGCTCAGGAGCTTGATGAGTTTGTACCACGGCTTGGCCTCAT

CAAGGCCAGCCCTGATGGCTTTCACTCCAGTGGCCAGTTCTTCAAATGCAGACACCAACC

TGTTGAAGTCGGGTCCGTGTTTTGTTGACATGTCCTCTTGCATTTGGTTGATGGTCTCTA

CCAGCTTGGTGAAGTTCGACCTGACGTCGGAGAAGAAGAAGGGCCCAGGGTTGGACTCAA

CGTCCCCTGCCAACTTGAGCAGGTCAAAGTTGAGGAACTGCTTTGCGGGCGCGATGATCT

TCTGCTTGTGTCTGTCTGCCGTGACCTCCACTGATAGTAGCGGTCTGGGGCAGTAGAGTT

CAGCCCGCTTCATGCGCACGAGAAGCTCTTGGATGCTTTCAGCACGCAGCGCCCCATAGT

TGAAGGACGAAGGGAGTTGCACGGCGACTCTCGCTGCAAGGGCCCCCATGTCGCCTCGTC

TGGGCGAAGCACCCGCTGAGTACTTGCTCGTCCCGTTGTACACTGTTGCCAACACGCGGT

GCGGCGCAGTGTAGGGGAGTGCAAGTCTCGTGAAGGGCGCTTTGAGGTATGCTGTGGGAT

TACCCGTATTAGCAAGAGCTGCCTCCGGGGCGCCGTTGGGCACCCATGTCAAGTTCCCCT

GGTGCTTTACCACAACTTCCAAGTCAGAAAAATAGTAGGTGGCTGAACGCAACAACGCAC

CTACCAACCCGTGCTGGTGAGTTTGCATGAGGTCTATGACGTGTGTGGGAGTGATGCTGT

TCACTTTCACAAACCTATCCATTATGAAGCCAACGTCCGTGTGGTGTCGCCTTTGGACCT

GTGTTTCACCGCCGTAGTTCTCCACAGTGGCGGTGACCGGGTCTGCAGATTCCCCTGTTG

CAGTGGTTTGCGCGCGAGGGTCAACGGGCAGGCGCAATTCAAAGTCTTTGCCGGCGCTCA

CCGACACGACCAGGGTGTCGTTCTCGGCCTTCCCGTGTGTGATTTGGTAAATGCAGACCC

ACCCCTGTACATTAGTTGTCTCCGCCACGTCTGACGCGGTATATGCGTAGTCTGCGGCGG

ACATGTACGGGATGGAGAAAGTGAACTTGGAATTCAGTCCTGTGTCCCACTCAGCATGGA

TGCAGTGGGCAGCTCCCTCAGGTGTTTCAGGTGGGGATTCCACGCCGGGTGGAATGTAGG

CTACCATATAACGGGCTTTTGAGTCAGTGGAACCAGTAAACATGAAGTGCAGGTTGATGG

TGCCCGAGTACTGTGCGTAGTACTGCGCGAGCCCCGAGAGGTAGGTGTTGGACATGCACT

TTGCGGCAAGGGAAACATCAAATTTGGCCAGAAGCCGTGTCTCGTCTGTCCTAGTAACAA

CGTACGGTCTCCCGTCGTCGAAACAGAGGAAGGTGGGACAAGCTTCAGCCACATCCAAGA

AGTTTGTGAAACGCCCCGGGTAGTTGTTTCTGGGCGGGTTGTACACTTTACCGTACACAG

GGTCAGCTGTTTTCGGGTCAGTTGTCACCAAACCACCATAGCCATCCGCACAGGCAACTG

GGACGATCCCCTCTTTCGAGGGGAGCTCCCCAGCCACGTGTACGTAGGTTGGGGCGATGT

TGGCGTAGACCTTGATCTGTGCTGCAGAGGTGGCACTGACGGTGAGCGGCGACACCACCA

TAACCACCAGTGTCCAGGGCTTGTGCTTCTTGTACTGGTCATACCTGTTCACACCAAGGT

AAGGCACCGTGATGTGTGCTGTCATGTTTGTCCTGGGGCTAATGAACTGGTGTGGGAAAA

GGGTGAGTTGGTATTTGTCACGCGTGCTCATGTCCTTCCACTCCGGGACCATGGCCACCA

GGAGACACCCACCGTTGAATTGGTTTCCAACGGCAGACACCTCCACGTCCCAGCCGTTCC

TCATGTACGCGTATGAGTTCACTAGGTGTCCGTAGACACCCGTGTGGTCGGTGGGGAGTT

CAAGTTTTTCCAGGTGTCCAAAGGCCTTGTCCGAGGTCCAGTCAAATAGATGTTTCTTGA

AGAACCTTTCTGCTTGTACCACCCGCGTCTCCAAGCCCGAGGTGTTGGGCCCGTTGACGT

GGTCCTCTCCAGTAGAGTACCCGTACGTGACCCCCACGCTCGACTGGGTTGTCGAGGTGG

TGTGTCCGTTGCGAGTGGTGAGGATGCGATCCTCCAGAAGAGTGGTCTCTTCAGTTTTCT

TGTCAGCAAGCAGTGCGCCAAACAAACCTGTGAATGCCGAGCTTGCAAGTTTGGAGAACC

AATCATTGTTTTGGGTGGTGCTTGTGTGGGTTGAAGTTGTGTCCGTGGAACCTTCATTAG

ACCCGCCACTAATGGCGTTGTCACCAAGCTGGGTGTCCATTGAGTTTTGGTACTGTTGCA

TGTAGTAGTTGTTTATAATGCTGCCAGTGTTGCCTGACTGGTTCTGAGACCCAGTGGCCG

GACTGGATTGCCCGGCCCCCTTGACGTACGCGGTCGCTTTCACAATCGCGTCACCGTTGA

ATGGCTCCATATCGTACGGGACAAAACACAGGACATCAGACGGATCTGGAGTCCACGGGT

AGAAGTCCTCGTCATCAATGGCGTACCACCCATTGGAGGTCAGACAGGCGAACACGGCAT

GTTCAGTACCCTTAAGGAAGATTCCTGCATGGAAATCAGCAAGGCACATGTCGGTACCGT

CGACCATGCACACCTCACTGGGTCGTGAGGCAGTGCCGATCCCAGTGTACAGCAAGTGCC

TGATGTTCCAGATTACGAGAGCGGGTGGCCCACCCTCGTGCAACTCCAGCCCAGTGATTC

TCTCTAGTTGTTCAATTGCCTGAAGAGTAAGATTCTCGGGCGAGTTGTAGACCCAGTCGA

AGAACGGTTCATCGACGTACCTAAACAGCTGCAGGATGGTGTTCAGCCAGCAGTTGTCGT

GGTTGTTGGGTCTAGAGTAAAATGTCTTTTTCTCACCGTTGTAAAGTGTGAATTCCATTT

TCTTTGATCCTCTAAACAGCAGCTTTATTTCTCTGATAGCCAATGCCAAAGCGGTAAAAC

AGTCAGTCGTGTTCATAAACGGTTTAGTGTTTAAAGAAAAGGTGCCGGCCTCCGGTCACC

TATTCAGGCATAGAAGCTTTTTAAACTGAGCACTTTGAAGAAGTCCCAGTCCCCTTCTCA

GATCCCGAGTGTCTCTTGTTACCTCGGGGTACCTGAAGGGCATCCTTAGCCTGTCTCCAG

GAACCAAGTACCAGTCTCAGTGTCACCTTAGAGCGTGTTTCGCAACAGTGCCGCCGTGCT

GGGGTTGCACACATGCAAGGTTCAGTGAACGTGGCTTTTGGCCCCGCGGGTCCTTGTTAC

CAAGGGGGAGTTCCCGCGGCTTGCATGCTCCGCTACGAAGCGACAAAGTGGTTACTAGCG

CCCGCCAGTGGACCAATCGTGGAGTTGGATCCAGTACAAAGTGTCGTCTCTCTAGACCTG

GAAAGACCAGGCGAAACTTCAAATTGCACGAGTGTGTCAGTTGAGGAAACCTGCTTGAGA

TCGTGTTTGTACAAGTGTTCCACAAGCGACTGCGCGTTTCGTGCGCTGACGTCCCATTTC

TTGAAAACGGGCAAAAGTGTGTTGTTGTGGGTGTTTCACTTCCCCCGGGAAGGTATATCG

TGTGGTTGCAATCCGTTAACGTCGGAAAAGACGGAGGAAAGCGTTTGGTTACAGTCCTTT

AAACGTCGGACA

>SAT2/CAR/DMS3/3/2019_PROBE_ENRICHMENT_CONSENSUS

AAGGATGCCCTCCAGGTACCCTGAGGTAACAAGCGACACTCAGGATCTGAGGAGGGGACT

GGGACTTCTGTAAAAGTGCCCGGTTTAAAAAGCTTCTATGCCTGAATAGGTGACCGGAGG

CCGGCACCTTTTCCTTGAACACACACTACTACATGAACACAACTGACTGTTTTATCGCTT

TGGTACAAGCTATCAGAGAGATCAAATTTTTGTTCAAACACACCAGAAAGATGGAACTCA

CGCTGCACAACGGCGAGAAGAAGACTTTCTACTCAAGGCCCAACCACCACGACAACTGCT

GGTTAAACACCATACTGCAGCTGTTCAGGTATGTCGATGAACCATTTCTCGACTGGGTCT

ACAACTCGCCTGAAAACCTCACGCTCCAAGCAATCGAACAGCTCGAGGAGCTCACAGGCC

TCAGCCTGCGCGAGGGTGGCCCTCCCGCCCTCGTGATTTGGAGCATCAAGCACTTGTTGT

ACACCGGGATCGGCACAGCCTCACGACCCAGCGAGGTGTGCATGGTCGACGGCACCGACA

TGTGTCTTGCTGACTTCCACGCAGGAATTTTTCTCAAGGGTACTGAACACGCAGTGTTCG

CCTGTTTGACCTCCAACGGGTGGTACGCCATCGACGACGAGGACTTCTACCCGTGGACTC

CGGACCCGTCCGATGTCCTGTGTTTTGTCCCGTACGACTTGGAACCATTCAACGGAGACG

CGGTTGTGAAAGCGACCGCATACGTGAAGGGAGCCGGGCAGTCCAGCCCGGCCACTGGAT

CACAAAATCAGTCAGGCAACACTGGTAGCATTATTAACAACTACTACATGCAGCAGTACC

AGAACTCAATGGACACACAGCTTGGTGACAACGCTATCAGTGGTGGCTCCAACGAGGGGT

CGACAGACACCACGTCGACACACACAAATAACACGCAGAACAACGATTGGTTCTCTAAAT

TGGCTCAATCCGCCATATCAGGGCTCTTCGGGGCGCTTTTGGCAGACAAGAAAACAGAAG

AGACCACGCTGTTGGAGGACAGGATCCTGACCACGCGTCACGGAACCACGACCTCCACCA

CACAAAGTTCTGTAGGTGTGACATTTGGCTACGCTGATGCTGATTCGTTTCGCCCGGGAC

CTAACACTTCCGGGCTTGAGACACGTGTGCAACAGGCAGAACGCTTCTTCAAGGAGAAGC

TCTTTGACTGGACCAGCGACAAACCTTTCGGCACGCTTTACGTGTTGGAGTTGCCCAAAG

ACCACAAGGGTATTTACGGTAAACTTACCGACTCCTACACGTACATGCGCAACGGCTGGG

ACGTCCAGGTCAGCGCAACCAGCACGCAGTTCAACGGCGGGTCACTGCTCGTAGCAATGG

TCCCAGAGCTGTGTAGCCTGAAAAGCAGAGAAGAGTTCCAACTCACCCTCTACCCACACC

AGTTCATTAACCCGCGTACCAACACAACTGCACACATACAGGTTCCATACCTGGGTGTGA

ACAGACACGATCAGGGTAAGCGCCACCAGGCGTGGTCTCTGGTTGTGATGGTGCTCACGC

CTCTTACAACCGAGGCTCAGATGAACAACGGCACCGTCGAGGTGTACGCTAACATCGCAC

CAACAAACGTAGTTGTGGCGGGCGAGCTGCCAGGTAAACAGGGTATTGTACCAGTTGCAG

CTGTCGACGGTTACGGCGGTTTCCAAAACACCGATCCGAAAACGGCTGACCCCATTTACG

GGCACGTGTACAACCCATCCAGAAACGACTGCCACGGGCGATACTCCAACCTTTTGGACG

TCGCCGAGGCGTGCCCAACGTTCCTGAACTTTGATGACAAGCCTTACATTGTGACCAAGA

ACAACGGTGACAAGGTGATGGCGACCTTCGACGTTGCTTTCACACACAAGGTACACAGGA

ACACGTTTCTGGCAGGCTTGGCCGATTACTACACACAGTATTCTGGCAGCCTAAATTACC

ACTTCATGTACACTGGACCCACACACCACAAGGCAAAGTTCATGGTGGCGTACGTGCCCC

CTGGCGTGGCGGCCGGGCAGCTGCCCAACACGCCAGAGGACGCCGCGCACTGCTACCATG

CGGAATGGGACACTGGATTGAACTCTTCTTTCTCGTTCGCAGTACCCTACATCTCCGCTG

CGGACTTTTCTTACACCCACACAGACACACCGGCCATGGCCAACATCAACGGCTGGGTGG

TTGTACTGCAGGTCACCGACACGCACTCTGCTGAAGCTGCTGTTGTGGTGTCAGTCAGCG

CGGGGCCAGACTTGGAATTCCGATTCCCCATTGACCCTGTGCGGCAGACTACCTCAGCGG

GAGAAGGCGCAGACGTCGTCACCACTGACCCATCCACACACGGTGGGAACGTTCGGGAAG

GCCGACGCAAACACACCGAAGTTGCGTTCCTTCTTGACCGCAGTACACATGTCCACACGA

ACAGGACATCTTTTGTTGTGGACCTCATGAACACAAAGGAGAAGGCACTCGTGGGCGCAA

TCCTGCGGGCTTCCACTTACTACTTTTGTGACCTTGAAATTGCATGTGTGGGCGAACACA

CAAGGGTCTTTTGGCAGCCTAACGGGGCGCCGCGGACTACCCAGCTTGGTGACAACCCCA

TGGTTTTCGCTAAGGGCGGTGTGACCCGCTTTGCCATCCCGTTCACGGCCCCACACCGGC

TGTTGTCTACTGTCTACAACGGTGAGTGCAGCTACCACAAGGCCGCCACTGCCATTCGTG

GGGACCGCGCAGCACTCGCGGCAAAATATGCTGGCAACACGCACACTTTGCCGCCAACCT

TCAACTTCGGGTTCGTGACCGCCGACAAGCCAGTCGATGTTTACTACCGAATGAAGAGGG

CTGAGCTGTACTGCCCACGCCCACTGCTGCCGGCCTACGACCACGCGGGCAGAGACAGAT

TTGACGCGCCCATCGGCGTCGAGAGACAGACCCTGAACTTCGACCTGTTGAAACTGGCGG

GAGACGTTGAGTCCAACCCTGGGCCCTTCTTCTTCTCCGACATCAGGTCCAATTTCACCA

AACTGGCAAGCGCCATCAGCCAGATGCAAGAGGACGTGTCCACAAAACACGGTCCTGACT

TTAACCGGCTGGTGAATGCATTTGAGGAGCTGACCCAAGGAGTTAGAGCCATTAGGGACG

GTCTTGACGAGGCCAAGCCCTGGTACAAAGTAATCAAACTCCTCAGCCGTTTGTCGTGCA

TGGCCGCTGTAGCAGCACGGTCCAAGGACCCGGTCCTTGTGGCCATCATGCTGGCTGACA

CCGGCCTTGAAATCCTGGACAGCACATTTGTCGTGAAGAAAATCGCCGACTCGCTCTCCA

GTGTTTTCCACGTGCCGGCCCCTGTCTTTAGTTTCGGAGCCCCGGTGCTATTGGCAGGTT

TGGTCAAGGTCGCCTCGAGCTTCTTCCGGTCAACACCCGAGGAACTTGAGAGAGCTGAGA

AACAGCTCAAGGCACGTGACATTAACGACATTCTCGCCATCCTCAAGAATGGTGAATGGC

TGGTCAAACTCATCCTGGCTATCCGCGACTGGATCAAGGCCTGGATCTCCTCAGAAGAGA

AGTTTGTTACCATGACAGACTTGGTGCCTGGTATTCTGGAGAAACAGCGGGATCTTCACG

ACCCCTCCAAGTACCATGAGGCAAAGGAGTGGCTTGAGAACGCCCGCCAGACGTGTCTCA

AGAACGGCAACATCCACATTGCTAATCTCTGCAAGGTCACAGCGCCCGCACCGAGCAAGT

CGAGACCTGAACCTGTGGTCGTCTGCCTCCGCGGCAAATCTGGCCAAGGTAAGAGTTTCC

TTGCTAACGTGCTTGCGCAGGCCATCTCCACCCACTACACTGGCAGAACAGACTCGGTAT

GGTACTGCCCACCTGACCCCGACCACTTTGACGGTTACAACCAACAAACCGTCGTGGTCA

TGGACGACCTCGGACAGAATCCCGACGGTAAGGACTTCAAGTACTTTGCCCAAATGGTCT

CCACAACCGGGTTTATCCCGCCCATGGCATCACTCGACGACAAGGGAAAACCATTCAACA

GCAAGGTCATCATCGCGACCTCCAACTTGTACTCTGGGTTCACACCACGTACAATGGTTT

GCCCTGAAGCACTGAACCGCAGGTTTCACTTTGACATCGACGTGAGCGCTAAGGACGGAT

ACAAAATTGGCAACAGATTGGACATTGTCAAAGCACTTGAAGACACCCACACAAACCCGG

TAGCAATGTTCCAGTACGATTGTGCCCTTCTGAACGGCATGGCTGTCGAGATGAAGAGAC

TGCAACAAGACTTGTTTAAGCCCCAACCACCGATCCTCAACGTGTACCAACTCGTAGACG

AGGTGATTGAGAGAGTTAGTTTACATGAGAAAATTGCCTCCCAACCTATTTTCAAACAAA

TTTCACTTCCTTCCCAAAAGTCTGTGCTCTACTTTCTCATTGAGAAGGGACAGCACGAAG

CTGCAATTGAATTCTTCGAAGGAATGGTCAGTGACTCCATCAAGGAGGAGCTGCGCCCCC

TTCTCCAACACACTTCATTTGTAAAACGCGCTTTCAAGCGCCTGAAGGAAAACTTTGAAA

TTGTTGTTCTGGTGTTGGCCCTGCTGGCCAATGTCATCATCATGATCCGCGAGACTCAGA

AGAGACAGGAAATGGTGGACGCTGCTGTGAACGATTACATCGAACGCACCGGTGTCACTA

CCGATGACCAGACTCTGGATGAGGCGGAGAAGAACCCTCTGGAAACGACCGGTGCCAGCA

CCGTCGGCTTCAGGGAGAGACTCCCCCCGCAGAAGGCAGACAATGACGTGAACTCCGAGC

CTGCTGAACCCGCGGAGGGAAAACCACATGCTGAAGGACCCTATGCCGGACCAATGGAAC

ACCAGAAACCTCTGAAAGTGAAGGCTAGACTGCCACAACAGGAGGGGCCCTACGCTGGTC

CGGTAGAGAGACAACAACCACTAAAGGTGAAAGTGAAACCCCCCGTCGTGAAGGAAGGAC

CTTACGAGGGGCCAGTGAAGAAACCTGTCGCTTTGAAAGTGAAAGCCAAGAATGTGATCA

TCACGGAGAGTGGAGCGCCACCCACCGACTTGCAGAAGATGGTGATGGCCAACACCAAGC

CAGTCGAGCTCATACTCGACGGGAAGACAGTGGCAATCTGCTGTGCCACTGGAGTGTTCG

GGACTGCCTATCTCGTGCCTCGTCATCTTTTCGCTGAGAAGTATGACAAGATCATGATTG

ACGGCAGGGCCGTGACAGACCGTGATTTCAGAGTGTTTGAGTTTGAGATCAAAGTAAAAG

GACAGGACATGCTCTCAGACGCTGCGCTCATGGTGCTGCACCGTGGGAATCGCGTGAGAG

ACATCACGAAGCACTTTCGTGACCAAGCCAGAATGAGAAAAGGAACCCCCGTGGTCGGTG

TAATCAACAACGCCGACGTCGGGAGACTCATCTTCTCTGGAGAGGCACTCACCTACAAAG

ACATTGTAGTGTGTATGGATGGTGATACCATGCCCGGCCTCTTTGCCTACAGAGCCGCCA

CCAAGGCCGGCTATTGTGGAGGAGCCGTTCTCGCGAAGGACGGAGCCGAGACTTTCATCG

TCGGCACTCACTCCGCAGGAGGAAACGGAGTTGGATACTGCTCTTGTGTTTCCAGGTCCA

TGCTCCTGCAGATGAAGGCACACATCGACCCCGAACCACACCACGAGGGGTTGATCGTGG

ACACCAGAGATGTGGAAGAGCGTGTGCACGTCATGCGCAAAACCAAGCTTGCACCCACCG

TTGCACACGGTGTGTTCAAACCTGACTTTGGGCCTGCTGCGTTGTCCAACAACGACAAGC

GCCTGGATGAAGGGGTCGTCCTCGACGACGTCATCTTCTCCAAACACAAGGGAGACACGA

AGATGTCAGAGGAAGACAAAAAGCTGTTCAGAGCTTGTGCTGCCGACTACGCGTCGCACC

TGCACTCAGTTCTTGGCACAGCAAATGCCCCATTGAGCATCTATGAGGCCATCAAGGGCA

TCGACGGGCTCGATGCCATGGAGCCTGACACCGCTCCTGGCTTGCCCTGGGCCACCCAGG

GGAAACGCCGCGGAGCCCTCATTGACTTCGAGAACGGCACTGTCGGACCCGAGGTTAGAG

AGGCGCTGAACCGCATGGAGAAAAGAGAGTACAGATTCACATGCCAGACATTCCTGAAAG

ACGAAATTCGTCCGATGGAAAAAGTTCGCGCCGGCAAGACTCGCATTGTCGATGTCTTGC

CTGTTGAGCACATTCTTTACACCAGAATGATGATTGGCAAGTTTTGTGCACAAATGCACT

CCAACAACGGACCGCAAATTGGCTCGGCGGTCGGCTGTAACCCAGATGTTGATTGGCAGA

GATTTGGAACCCACTTTGCCCAGTACCGGAACGTGTGGGACGTGGATTATTCGGCCTTTG

ATGCTAACCACTGTAGTGATGCCATGAACATCATGTTCGAGGAGGTTTTCCGCCCGGAGT

TTGGTTTCCACCCTAACGCGGAGTGGATCCTGAAGACTCTGGTCAACACGGAACATGCCT

ACGAGAACAAGCGCATTACGGTGGAAGGTGGTATGCCCTCGGGTTGTTCCGCCACCAGCA

TCATCAACACAATTCTTAACAACATTTACGTGCTCTACGCCCTGCGTAGACACTATGAGG

GGGTCGAGCTGTCTACCTACTCGATGATTTCCTACGGGGATGACATCGTGGTGGCAAGTG

ACTTTGACCTGGACTTTGAAGCTCTCAAGCCTCACTTCAAGTCCGTTGGCCAAACTATCA

CTCCAGCCGACAAAAGTGACAAAGGTTTTGTACTTGGTCAGTCCATTACCGACGTTACTT

TCCTCAAGAGGCACTTCCACATGGATTATGGAACTGGGTTTTACAAACCTGTGATGGCTT

CGAAGACCCTCGAAGCTATCCTCTCCTTTGCACGCCGTGGGACCATACAGGAGAAGTTGA

CCTCCGTGGCAGGACTCGCCGTCCACTCCGGGCCAGACGAGTACCAGAGACTCTTCGAGC

CTTTCACCGGGCTCTTTGAGATTCCTAGCTACAGGTCACTTTACCTGCGTTGGGTGAACG

CCGTGTGCGGTGGCACATAATCCCCTAGCTTCCTACCAAGGACACTAGGGCGAGTGTCGC

CGTTGGAGTGAAAGGCCCGAAAGGACCTTTTCCCGCTTCCCTGATTCAAAAAAAAAAAAA

AAAAAAAAAAAAAAG
